# ^1^H–NMR Metabolomic Biomarkers of Poor Outcome after Hemorrhagic Shock are Absent in Hibernators

**DOI:** 10.1371/journal.pone.0107493

**Published:** 2014-09-11

**Authors:** Lori K. Bogren, Carl J. Murphy, Erin L. Johnston, Neeraj Sinha, Natalie J. Serkova, Kelly L. Drew

**Affiliations:** 1 Department of Chemistry and Biochemistry, University of Alaska Fairbanks, Fairbanks, AK, United States of America; 2 Institute of Arctic Biology, University of Alaska Fairbanks, Fairbanks, AK, United States of America; 3 Centre of Biomedical Research, SGPGIMS Campus, Lucknow, Uttar Pradesh, India; 4 Department of Anesthesiology, University of Colorado Anschutz Medical Campus, Aurora, CO, United States of America; 5 University of Colorado Cancer Center, University of Colorado Anschutz Medical Campus, Aurora, CO, United States of America; National Institutes of Health, United States of America

## Abstract

**Background:**

Hemorrhagic shock (HS) following trauma is a leading cause of death among persons under the age of 40. During HS the body undergoes systemic warm ischemia followed by reperfusion during medical intervention. Ischemia/reperfusion (I/R) results in a disruption of cellular metabolic processes that ultimately lead to tissue and organ dysfunction or failure. Resistance to I/R injury is a characteristic of hibernating mammals. The present study sought to identify circulating metabolites in the rat as biomarkers for metabolic alterations associated with poor outcome after HS. Arctic ground squirrels (AGS), a hibernating species that resists I/R injury independent of decreased body temperature (warm I/R), was used as a negative control.

**Methodology/principal findings:**

Male Sprague-Dawley rats and AGS were subject to HS by withdrawing blood to a mean arterial pressure (MAP) of 35 mmHg and maintaining the low MAP for 20 min before reperfusing with Ringers. The animals’ temperature was maintained at 37±0.5°C for the duration of the experiment. Plasma samples were taken immediately before hemorrhage and three hours after reperfusion. Hydrophilic and lipid metabolites from plasma were then analyzed via ^1^H–NMR from unprocessed plasma and lipid extracts, respectively. Rats, susceptible to I/R injury, had a qualitative shift in their hydrophilic metabolic fingerprint including differential activation of glucose and anaerobic metabolism and had alterations in several metabolites during I/R indicative of metabolic adjustments and organ damage. In contrast, I/R injury resistant AGS, regardless of season or body temperature, maintained a stable metabolic homeostasis revealed by a qualitative ^1^H–NMR metabolic profile with few changes in quantified metabolites during HS-induced global I/R.

**Conclusions/significance:**

An increase in circulating metabolites indicative of anaerobic metabolism and activation of glycolytic pathways is associated with poor prognosis after HS in rats. These same biomarkers are absent in AGS after HS with warm I/R.

## Introduction

Hemorrhagic shock is a life threating medical condition most often associated with trauma. Trauma is the third leading cause of death for persons under the age of 40 with approximately one third of these deaths resulting from HS [1], [2]. During HS the massive loss of blood reduces systemic blood pressure and produces I/R injury. Hypoperfusion of tissue decreases the removal of waste products and supply of oxygen and nutrients resulting in cellular dysfunction. This is then compounded with the generation of reactive oxygen species during reperfusion that leads to further tissue damage [3], [4].

Hibernating animals, such as ground squirrels, have been shown to have an innate resistance to I/R injury. This resistance is found not only in isolated organ I/R, but also in systemic I/R such as HS [5]–[11]. The arctic ground squirrel (*Urocitellus parryii*, AGS) survive HS-induced I/R without tissue damage or multi organ failure, independent of their hibernation state, season, or tissue temperature [11]. There is also a well-documented ability in ground squirrels to switch fuel sources from carbohydrate- to lipid-based metabolism during their hibernation season [6], [12]–[22]. Previous studies in our lab indicated that, in conjunction to their resistance to HS-I/R injury, AGS also have an altered plasma glucose/lactate ratio and base excess response during HS as compared to an I/R injury-prone species, indicating that they may have a different metabolic response to I/R [11]. Differences in metabolic process during I/R in the injury-prone species versus an injury-resistant species is not known and may provide valuable information as to the molecular basis of the protective phenotype.

This study sought to identify circulating metabolites in the rat as biomarkers for metabolic alterations associated with poor outcome after HS. AGS, a hibernating species that resists I/R injury independent of temperature drop (warm I/R), was used as a negative control. Specifically, we expected to see differential activation of glucose and anaerobic metabolism in the I/R injury prone rat and not in the I/R injury-resistant AGS. Another purpose of this study was to identify changes in circulating metabolic profiles and metabolites in hibernators which may contribute to their innate resistance against I/R injury and muli organ failure. Refinement in the identification of these metabolic pathways will provide further insight into this mechanism. We found that during HS-I/R rats displayed a shift in metabolic profiles related to carbohydrate turnover, anaerobic metabolism, and organ dysfunction while AGS maintained their metabolic homeostasis and lacked biomarkers of organ dysfunction.

## Methods

^1^H–NMR-metabolomic analysis of total plasma samples and their lipophilic fractions taken before and after HS-I/R was conducted for I/R injury prone rats as well as I/R injury resistant summer/euthermic (EU) and winter/interbout arousal (IBA) AGS. Spectral patterns were qualitatively compared for overall changes in the metabolic “fingerprints” for naïve, sham, and hemorrhaged animals. Qualitative analysis was followed by quantification of specific metabolites identified in the spectra. These parameters from each experimental condition were then compared between the I/R injury-prone rat and the injury-resistant EU-, IBA-AGS.

### Animals and ethics statement

All animal procedures were performed in strict accordance with the *Guide for the Care and Use of Laboratory Animals* and approved by the Animal Use and Care Committee of the University of Alaska Fairbanks. Capture and holding of AGS was performed under permit by the Alaska Department of Fish and Game. Juvenile AGS were live trapped north of the Brooks Range (66°38′N, 149°38′W) in Alaska during July and were maintained in captivity at least ten months prior to the experiments. The AGS were housed at 22°C under light conditions based on 69° latitude from time of capture until late August when they were then transferred to a cold chamber. AGS remained in the cold chamber (2°C) with 4∶20 light∶dark conditions until the experiments which were conducted between May and June (summer, euthermic) and December- February (winter, interbout arousal). Food and water were provided ad libitum but to normalize glycogen levels, food was removed twenty hours prior to surgery. Both male and female AGS (438–1123 g, n = 41) were used in this study due to the availability of wild caught animals. There is not an opportunity to select the gender of wild-trapped AGS. Consequently, gender is balanced across groups. Because AGS are reproductive for a short time period in the early spring, animals were not used during the four week period following the end of spontaneous torpor bouts when estrogen and testosterone levels are elevated [25], [26]. In contrast, female rats have estrus cycles every 4–5 days [23] with concurrent fluctuations in estrogen levels. Estrogen is known to be protective against I/R injury [24] Thus, female rats were not used in this study to avoid the confounding effect of estrogen on I/R protection.

Summer/euthermic status of an animal was assessed by body temperature, activity and lack of spontaneous torpor for at least four weeks. Animals were considered to be in the winter/hibernation season when they had been having regular spontaneous torpor bouts for at least eight weeks prior to the experiments. For IBA animals, arousal was induced 18 hours before the start of the experiment by gentle calisthenics. All animals were at euthermic core body temperatures at the start of the experiment. Male Sprague-Dawley rats (350–420 g, n = 24) were obtained from University of Alaska Fairbanks Animal Resource Center (Sprague-Dawley derived from Simonson Laboratory, Gilroy, CA).

### Hemorrhagic shock

Anesthesia was induced and maintained with isoflurane and a 30∶70 mixture of O_2_ and N_2_O. Once unresponsive, the animals were intubated with an endotracheal catheter and connected to a ventilator humidified with mucomist (1.4 mL/70 mL H_2_O). Vital signs were monitored by electrocardiogram (EKG) and a rectal (core body temperature) and temporalis (head/brain temperature) thermocouples. The animals were then cannulated in their right femoral artery and vein and left femoral artery. MAP was measured via the femoral arterial catheter connected to a precalibrated pressure transducer and was recorded continuously. Blood was sampled every ten minutes for P_CO2_ and P_O2_. Mechanically ventilated stroke volume, breaths per minute, and percent O_2_ were changed until blood gases were within predetermined normal ranges as previously described [9], [10]. Core body temperature and head temperature were maintained at 37±0.5°C from the start of surgical preparation until post HS monitoring. This was achieved with a heated water blanket under the animal and heat lamps placed above the head and back turned on below 36.5°C and off above 37.5°C by thermocouples placed in the temporalis muscle and lower bowel via T-C5C32 temperature controllers (Omega, Stamford, CT). Blood was then withdrawn over a 15 minute period to achieve a target MAP of 35 mmHg. The MAP was held at ∼35 mmHg for 20 minutes before animals were reperfused intravenously with non-lactated Ringers solution (148 mM NaCl, 2 mM CaCl_2_, 4 mM KCl) equal to 2/3 volume of the blood removed during hemorrhage (Hospira, Inc.,Lake Forest, IL). After HS animals were monitored for an additional three hours under anesthesia and then euthanized via decapitation. Sham hemorrhagic shock (SHS) animals were subject to the surgical procedure, but blood was not removed nor Ringers administered. Experimental design and groups are summarized in Figure S1 and Table S1. I/R and sham experiments were conducted on alternating days. Naive animals were included as a control for effects of surgery. AGS were matched for sex and hibernation phenotype (Tables S2-S5).

EDTA-treated plasma samples were collected from the femoral artery immediately prior to HS (baseline) and by cardiac puncture at the end of the three hour monitoring period. Blood from naïve animals was collected via cardiac puncture. Samples were kept on ice and then snap frozen in liquid nitrogen before being stored at −80°C.

### Quantitative ^1^H–NMR on unprocessed plasma and lipid extracts

For NMR analysis, samples of EDTA-preserved plasma in deuterium oxide (D_2_O, 99.8% Alfa Aesar, Ward Hill, MA; 150∶450 µL) were transferred into 5-mm NMR tubes (Wilmad Lab Glass, Buena, NJ). ^1^H–NMR spectra were acquired at 5°C (based on Methanol calibration) with a 600-MHz Bruker Avance-III system running TopSpin 3.0 software (Bruker Biospin, Fremont, CA) using a dual resonance high resolution SmartProbe with single axis Z-gradient. The water signal was suppressed using NOESY presaturation followed by CPMG relaxation editing for suppression of macromolecules (“PROF_CPMG” parameter set in TopSpin 3.0). A standard, trimethylsilyl propionic-2,2,3,3-tetradeuteropropionic acid (TMSP, 5 mM in D_2_O) contained in a coaxial insert and placed in the NMR tube was used for metabolite quantification of fully relaxed ^1^H–NMR spectra and as a ^1^H chemical shift reference (0.0 ppm). The ^1^H–NMR peaks for single metabolites were identified and referred to published chemical shift or a metabolite chemical shift library [27]–[30]. Two dimensional experiments including COSY, JRes, and HMQC were used to confirm assignments for selected samples (Fig. S2–S11). After Fourier transformation, phasing, and baseline correction in TopSpin, each ^1^H peak was integrated with Amix (Bruker Biospin). The absolute concentration of each metabolite was then referred to the TMSP integral and calculated according to the equation adapted from Serkova et al (25).: *C_x_ = (I_x_:N_x_xC)/I*:9; where *Cx* is metabolite concentration (µmol/mL), *Ix* is integral of metabolite ^1^H peak, *Nx* is number of protons in metabolite ^1^H peak, *C* is TMSP concentration, and *I* is integral of TMSP ^1^H peak at 0 ppm (this is nine as TMSP contains nine protons). An additional correction factor of 8.68 was applied to adjust for the differences in diameters between the NMR tube and the insert (derived as the ratio of the cross-sectional areas of the tubes). The final metabolite concentrations were expressed as mM of EDTA-plasma volume. Glucose concentrations were not determined from the plasma spectra as presaturation to diminish the water signal also affected glucose peaks that are in close proximity.

Lipids were extracted from the EDTA-plasma samples using a dual chloroform/methanol extraction. Briefly, plasma was mixed (1∶2 vol/vol) with cold chloroform/methanol (1∶2 vol/vol) and centrifuged. Liquid was removed from the pellet and the pellet was washed again in chloroform/methanol and centrifuged. The second supernatant was removed and combined with the first which together were washed with cold water. The mixture was centrifuged to separate the lipophilic and hydrophilic fractions. Lipid extracts were lyophilized and reconstituted in 0.6 mL deuterated chloroform/methanol (1∶2, vol/vol) for NMR analysis. TMP was contained in the deuterated methanol (0.3% 1.47 mM) and a coaxial insert was not used. No suppression of macromolecules was used.

### Statistics

Multivariate analysis (partial least squares-discriminant analysis; PLS-DA) was used for comprehensive metabolite profile analysis within complex ^1^H–NMR spectra. Initially, principle component analysis (PCA) was used but did not show good separation due to small sample size. Therefore, PLS-DA was selected for further analysis. PLS-DA was performed using MetaboAnalyst, a freely available, web-based application designed to support quantitative metabolomics [31], [32]. Before being analyzed, the spectra were segmented into “buckets” each with a 0.01 ppm width. The water region (4.8–5.2 ppm) and EDTA regions (2.53–2.58, 3.1–3.3, and 3.6–3.7 ppm) were excluded from the bucketing and multivariate analysis of the unextracted plasma samples while the chloroform (8.0–7.5 ppm) and methanol (5.22–4.76 & 3.42–3.25 ppm) regions were excluded from the lipid spectra. Before PLS-DA, data was normalized to a Gaussian distribution (a feature in MetaboAnalyst; http://www.metaboanalyst.ca).

All data are presented as mean ± SEM. Fold change was calculated as (final concentration - initial concentration)/initial concentration. Absolute individual concentrations of each metabolite were analyzed via three-way ANOVA with repeated measures (SAS; Cary, NC). Metabolites with a significant time × group (EU-AGS, IBA-AGS, or rat) × treatment (HS or SHS) interaction were further analyzed via two-way ANOVA with repeated measures. All significant effects in ANOVAs were followed by a Tukey’s post-hoc test. Statistical significance was considered to be a *p* value of <0.05.

## Results

### Metabolic ^1^H–NMR spectral pattern difference between test groups

**Figure 1:**
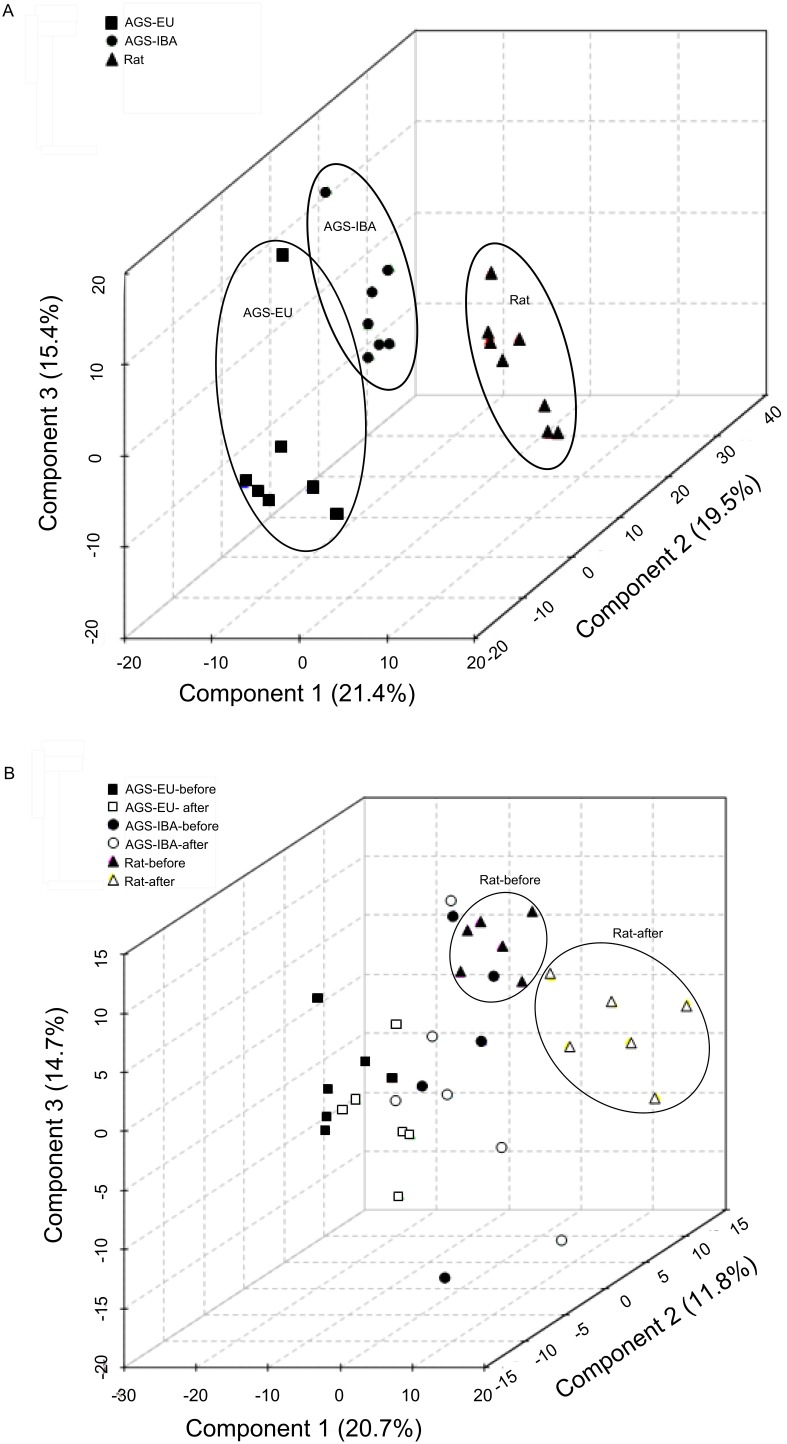
Hydrophilic metabolic profiles. A: Hydrophilic metabolic profiles for naïve summer euthermic and winter IBA-AGS tended to form distinct clusters separate from each other and the naïve rats. However, there was no statistically significant discrimination between the classes (R^2^ = 1.00, Q^2^ = 0.79, permutation p = 0.70) B: I/R injury–prone rats displayed different hydrophilic metabolic profiles before and three hours after hemorrhagic shock (R^2^ = 0.95, Q^2^ = 0.79, permutation p<0.01.). No such clustering was evident for the AGS in either hibernation season. PLS-DA analysis of ^1^H^–^NMR of naïve plasma samples (A) or samples from before and after hemorrhagic shock (B).

The global metabolic profile of circulating metabolites present in the plasma of naïve animals was assessed via ^1^H–NMR spectral pattern comparison between each of the test groups (rats, EU-AGS, and IBA-AGS) through the multivariate statistical analysis, PLS-DA. PLS-DA is a supervised classification method that maximizes the separation of groups of data based on variables in the data that are ranked on their efficacy in explaining differences in the groups. PLS-DA summarizes the original variables into fewer variables using their weighted averages. These are then cross-validated to select an optimal number of components for classification. The top three components are then used for separation with each one being an axis (x, y, and z). The percentage next to each component indicates to what degree it contributed to the separation [32]. When analyzed for spectral pattern similarities using PLS-DA, each naïve group had distinct clustering (Fig. 1A). The clusters of the EU-AGS and IBA-AGS were closer in proximity to each other than to the rats, indicating greater spectral similarity between the two seasonalities of AGS than either had with the other species. Most of the animals in the AGS groups clustered together; however, in each group there was a distinct outlier. The naïve rats showed less of this clustering and were more spread out. Each group (rat, EU-AGS, IBA-AGS) was examined with PLS-DA for differences between all experimental points (naïve vs. before HS vs. after HS vs. before SHS vs after SHS; Fig. S12). There were differences seen due to the surgery itself (before SHS vs. after SHS) and differences from naïve animals. To account for this, only the metabolites that changed after HS (and not after SHS) were analyzed. Due to the basal metabolic differences between species and hibernation season, metabolites that changed due the HS were normalized to baseline values and the fold change was analyzed.

**Figure 2:**
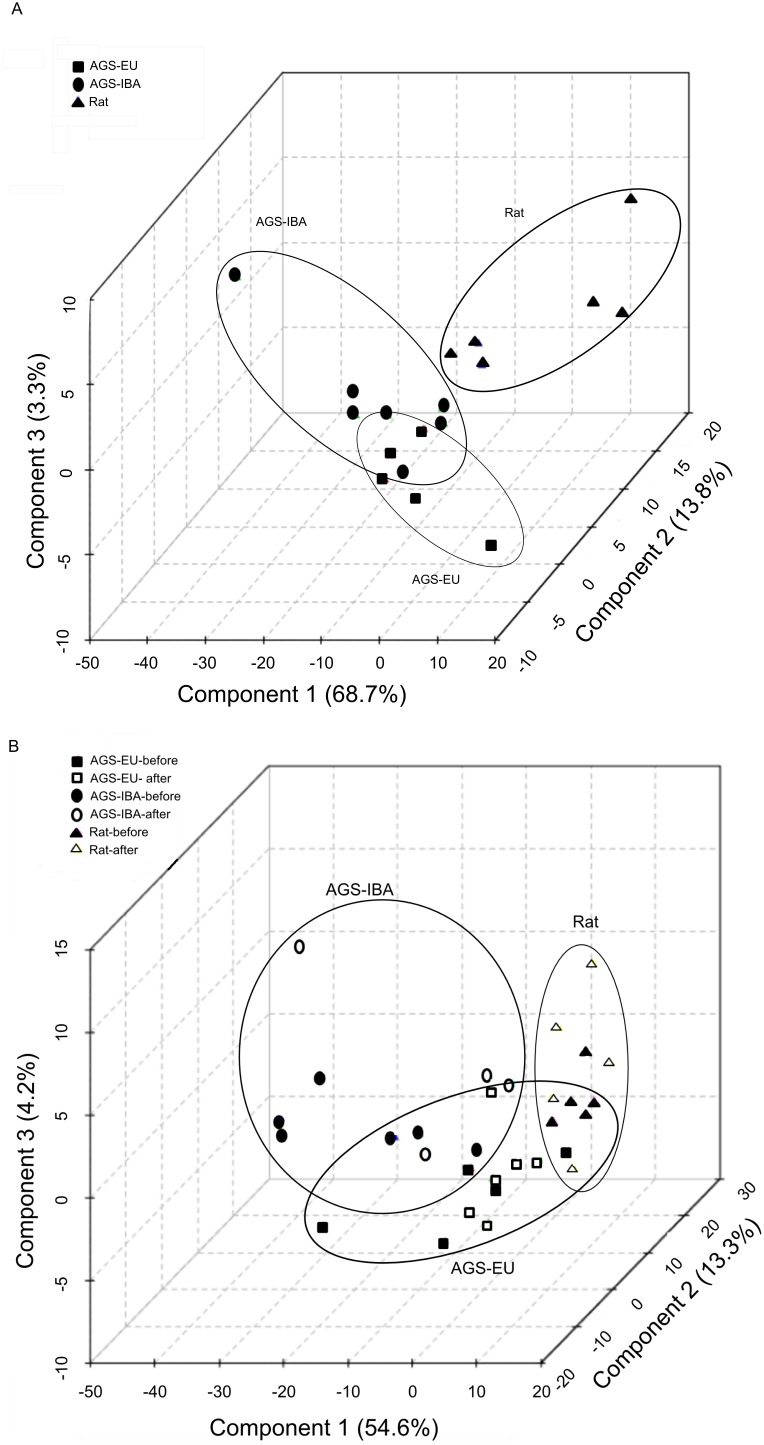
Lipid metabolic profiles. A: Lipid metabolic profiles for naïve rat tended to be distinct from summer euthermic and winter IBA AGS while both season of AGS overlapped via PLS-DA analysis of the ^1^H^–^NMR spectra (R^2^ = 0.75, Q^2^ = −0.10, permutation p = 0.12). B: Interbout arousal arctic ground squirrels tended to be distinct from I/R prone rats while euthermic arctic ground squirrels overlapped both rats and interbout arousal squirrels in PLS-DA analysis of ^1^H^–^NMR lipid-extracted plasma samples before and after hemorrhagic shock(R^2^ = 0.70, Q^2^ = −0.02, permutation p = 0.28). For the lipid metabolic profiles, there was not a statistically significant discrimination between the classes.

Rats from both time points formed two distinct groupings, one for the before HS samples and the other for the samples collected three hours after HS. This indicates that the rats experienced distinct alterations in the ^1^H–NMR spectra as a consequence of HS. In all test groups, PLS-DA analysis of the HS and sham animals’ spectra immediately before HS or sham treatment showed the same distinct clustering as observed in naïve animals (data not shown). When ^1^H–NMR spectra of plasma samples before and after hemorrhagic shock was analyzed via PLS-DA, clustering of both groups of AGS was distinct from rats. In contrast to rats, both EU- and IBA-AGS, before and after HS overlapped one another without any distinct, segregated clusters (Fig. 1B). We next looked at the ^1^H–NMR spectra of the non-polar molecules. When obtaining spectra of total-plasma, a CPMG relaxation editing sequence was used to suppress the macromolecule signals (including lipids) from obscuring the hydrophilic metabolite peaks. To evaluate the possible contribution of lipid metabolism in AGS resistance to I/R-induced multi organ failure, plasma samples were lipid extracted and PLS-DA was further employed to determine spectral pattern similarities in the non-polar fraction. PLS-DA analysis of the lipophilic components of the plasma samples was conducted on naive animals of each test group (Fig. 2A) and from samples taken before and three hours after HS (Fig. 2B). For the naïve animals, the lipid spectra for both the EU- and IBA-AGS were clustered together with the exception of an outlier in each group. The AGS clustering was distinct from the rats, which had a more scattered distribution. Lipid ^1^H–NMR spectra was also evaluated before and after HS (Fig. 2B). Unlike the naïve animals, those subject to surgical conditions and HS lacked any encapsulated clustering; the rats did not form a distinct grouping but overlapped with the EU-AGS. The EU-AGS grouping crossed into both the rat and IBA-AGS clusters, but the rat and IBA-AGS did not intersect. Within each test group, the spectra before and after HS did not segregate, indicating there was not a spectral pattern difference before and after HS in any of the three groups.

### Changes in circulating metabolite concentrations after hemorrhagic shock

**Figure 3:**
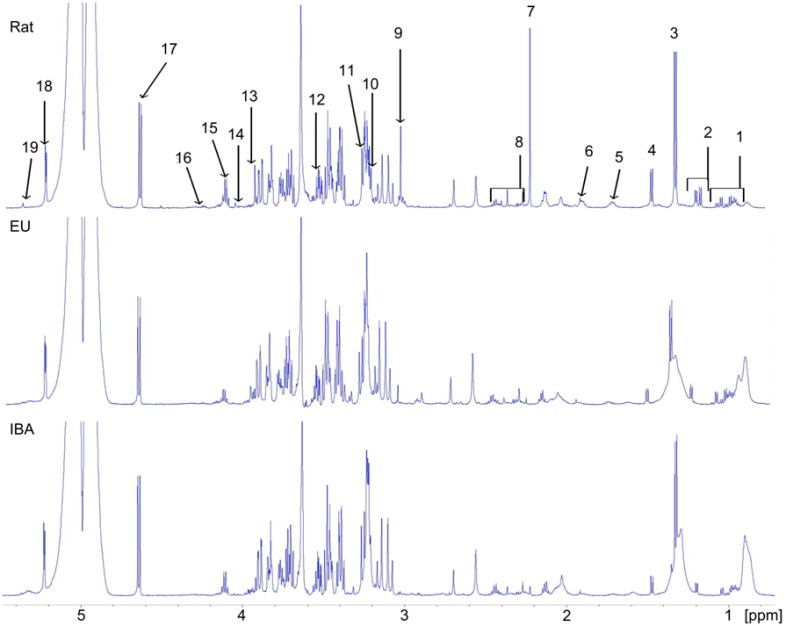
Representative ^1^H^–^NMR spectra of unprocessed plasma. Spectra from naïve rats, summer euthermic (EU) arctic ground squirrel, and winter interbout arousal (IBA) arctic ground squirrels. NMR peak assignments: 1, valine+leucine+isoleucine; 2, DBHB + isobutyrate; 3, lactate + threonine; 4, alanine; 5, lysine; 6, acetate; 7, acetoacetate; 8, glutamine + succinate + pyruvate + glutamate; 9, creatine + creatinine; 10, choline; 11, betaine; 12, glycine; 13, creatine; 14, creatinine; 15, lactate; 16, threonine; 17, β-glucose; 18, α-glucose; 19, allantoin.

**Figure 4:**
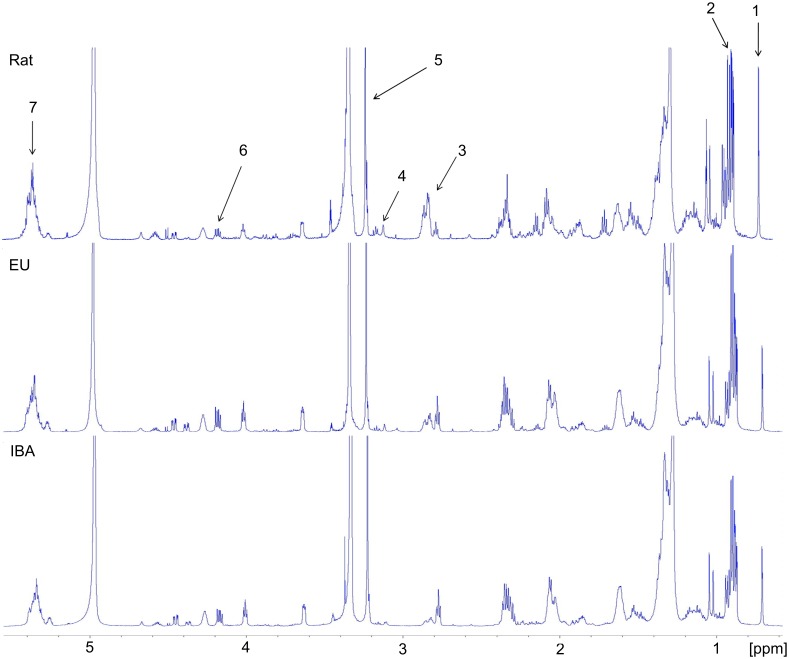
Representative ^1^H^–^NMR spectra of plasma lipid extract samples. Spectra from naïve rats, summer euthermic (EU) arctic ground squirrel, and winter interbout arousal (IBA) arctic ground squirrels. NMR peak assignments: 1, cholesterol; 2, total triglycerides; 3, PUFA, polyunsaturated fatty acids; 4, phosphotidyl ethanolamine; 5, cholines; 6, TAG, triacylglycerol; 7, UFA, unsaturated fatty acids.

Quantitative analysis of the metabolites identified in the ^1^H–NMR spectra was employed to obtain more specific circulating metabolic profiles of the rats, EU-AGS, and IBA-AGS and to determine any changes in the concentration during I/R. Analysis was conducted on the untreated plasma samples (Fig. 3) and on the lipophilic components of the lipid-extracted samples (Fig. 4). Figures 3 and 4 show representative spectra from naïve animals of each of the groups with peaks used to quantify specific metabolites identified.

A combined total of 32 metabolites were identified in the unprocessed plasma and lipophilic spectra with 25 being found in the unprocessed plasma spectra and seven being identified in the lipophilic spectra. Of the 32 metabolites, only four differed between the naïve rats and AGS (IBA-AGS and EU AGS; Table 1). These were acetate, choline, glutamine and threonine. Rats had higher circulating levels of all of these metabolites save choline, where the AGS had greater concentration. When focusing on changes during I/R, only eight (acetate, glutamine, β-hydroxybutyrate (DBHB), lactate, lysine, tyrosine, alanine, and histidine) had a significant change in concentration from before HS to three hours after HS that was different between group (rat, EU-AGS, or IBA-AGS) and was not found in the sham experiments (*p*<0.05, ANOVA, time × group × treatment; Table 2; naïve data, Table 1).

**Table 1:** Average concentrations (mM plasma) and SEM of metabolites in naïve animals for summer euthermic (EU) arctic ground squirrels, winter interbout arousal (IBA) arctic ground squirrels, and rats.

	Naïve EU-AGS	Naïve IBA-AGS	Naïve Rat	ANOVA p
Hydrophilic metabolites by ^1^H–NMR				
Acetoacetate	0.095±0.209	0.459±0.772	0.096±0.185	0.088
Acetate	0.110±0.009^a^	0.078±0.005^a^	0.190±0.013^b^	<0.0001
Alanine	0.454±0.086^a,b^	0.346±0.040^a^	0.625±0.040^b^	0.001
Allantoin	0.218±0.073	0.232±0.052	0.138±0.019	0.372
Betaine	1.004±0.151^a^	0.877±0.081^a,b^	0.570±0.043^b^	0.014
Choline	0.646±0.053^a^	0.726±0.045^a^	0.462±0.034^b^	0.001
Creatinine	0.125±0.209	0.105±0.178	0.111±0.164	0.834
Creatine	0.217±0.019	0.168±0.027	0.225±0.012	0.105
Formate	0.199±0.013	0.152±0.008	0.141±0.025	0.079
Glutamate	0.229±0.223	0.248±0.225	0.252±0.160	0.561
Glutamine	1.118±0.099^a^	1.098±0.093^a^	2.022±0.045^b^	<0.0001
Glycine	0.712±0.062	0.839±0.072	0.735±0.066	0.387
Histidine	0.076±0.007	0.086±0.006	0.066±0.006	0.112
β-hydroxybutyrate	0.442±0.095	0.668±0.240	0.161±0.016	0.061
Isobutyrate	3.591±2.932	0.129±0.218	0.170±0.410	0.321
Isoleucine	0.199±0.053	0.130±0.019	0.127±0.007	0.215
Lactate	1.808±0.219	1.943±0.144	2.227±0.222	0.335
Leucine	0.441±0.099	0.359±0.004	0.273±0.011	0.164
Lysine	0.461±0.387	0.380±0.279	0.470±0.186	0.181
Phenylalanine	0.074±0.009	0.063±0.008	0.090±0.009	0.106
Pyruvate	0.148±0.008^a^	0.108±0.010^b^	0.146±0.009^a^	0.011
Succinate	0.046±0.118	0.072±0.217	0.039±0.058	0.079
Threonine	0.433±0.047^a^	0.440±0.058^a^	0.608±0.037^b^	0.023
Tyrosine	0.156±0.024^a^	0.083±0.011^b^	0.116±0.014^a,b^	0.025
Valine	0.308±0.046^a^	0.214±0.014^a,b^	0.210±0.005^b^	0.030
Lipophilic metabolites by ^1^H–NMR				
TAG	10.687±6.183	11.682±3.544	1.903±0.340	0.211
Cholines	6.630±1.097^a,b^	11.547±3.239^a^	2.405±0.603^b^	0.032
PUFA	11.939±2.286	12.248±3.498	6.465±1.068	0.256
Total Triglycerides	64.594±30.525	84.074±22.556	15.428±2.157	0.108
Cholesterol	4.968±1.210^a,b^	9.417±2.144^a^	1.904±0.223^b^	0.010
Phosphotidyl Ethanolamine	1.080±0.244	2.167±0.579	1.492±0.348	0.230
UFA	51.794±23.843	64.400±17.948	15.193±2.647	0.190

Average concentrations (mM plasma) ± S.E.M. of metabolites for naïve summer euthermic (EU) arctic ground squirrels, winter interbout arousal (IBA) arctic ground squirrels, and rats. TAG, triacylglycerol; PUFA, Polyunsaturated fatty acids. n = 6–7 for all groups. ANVOA value and significant differences (p<0.05, Tukey) are indicated by differing superscript letters.

**Table 2:** Average concentrations (mM plasma) and SEM of metabolites for summer euthermic (EU) arctic ground squirrels, winter interbout arousal (IBA) arctic ground squirrels, and rats immediately before and three hours after hemorrhagic shock (HS) or sham (SHS).

Metabolite	ppm	Trt	EU-AGS				IBA-AGS				Rat		ANOVA *p*	Interaction or Main Effect
			Before		After		Before		After		Before	After		
Hydrophilic metabolites by ^1^H–NMR														
Acetate	1.91	HS		0.075±0.018		0.086±0.011		0.090±0.009		0.097±0.009	0.165±0.011	0.028±0.023	0.0009	time × × group × treatment
		SHS		0.079±0.011		0.099±0.006		0.075±0.006		0.082±0.011	0.153±0.010	0.170±0.023		
Alanine	1.47	HS		0.357±0.055		0.683±0.104		0.551±0.152		0.940±0.180	0.770±0.078	1.945±0.195	0.0007	time × group × treatment
		SHS		0.479±0.067		0.640±0.094		0.316±0.031		0.394±0.333	0.763±0.066	0.926±0.159		
Glutamine	2.44	HS		1.150±0.123		1.446±0.049		2.753±0.353		2.214±0.094	1.740±0.075	1.958±0.055	0.0086	time × group × treatment
		SHS		1.122±0.069		1.472±0.102		2.936±0.668		3.585±0.643	1.605±0.090	1.882±0.081		
Histidine	7.05	HS		0.073±0.010		0.090±0.008		0.070±0.007		0.082±0.010	0.059±0.007	0.064±0.019	0.0126	time × group × treatment
		SHS		0.086±0.008		0.094±0.011		0.085±0.007		0.067±0.018	0.068±0.003	0.115±0.007		
DBHB	1.20	HS		0.238±0.068		0.334±0.057		1.765±0.683		0.861±0.252	0.894±0.043	0.747±0.042	0.0329	time × group × treatment
		SHS		0.264±0.059		0.493±0.063		2.883±1.093		3.576±1.111	0.924±0.118	0.960±0.215		
Lactate	4.11	HS		1.768±0.235		3.402±0.611		2.291±0.231		3.674±0.421	1.665±0.069	7.573±0.431	<0.0001	time × group × treatment
		SHS		2.497±0.406		3.124±0.379		2.123±0.105		2.189±0.231	1.972±0.204	3.537±0.500		
Tyrosine	6.88	HS		0.069±0.009		0.109±0.026		0.035±0.008		0.037±0.008	0.054±0.007	0.201±0.036	0.0002	time × group × treatment
		SHS		0.060±0.005		0.074±0.013		0.050±0.005		0.034±0.007	0.058±0.003	0.058±0.009		
Lysine	1.71	HS		0.471±0.042		0.674±0.050		0.583±0.139		0.523±0.048	0.754±0.061	1.513±0.087	0.0032	time × group × treatment
		SHS		0.483±0.043		0.593±0.038		0.440±0.051		0.5234±0.048	0.677±0.072	0.838±0.166		
Succinate	2.40	HS		0.034±0.005		0.058±0.008		0.153±0.059		0.094±0.019	0.109±0.004	0.226±0.024	<0.0001	time × group
		SHS		0.033±0.004		0.054±0.006		0.340±0.106		0.2454±0.087	0.105±0.009	0.143±0.020		
Allantoin	5.37	HS		0.124±0.013		0.187±0.020		0.696±0.391		0.475±0.118	0.081±0.008	0.409±0.023	0.0405	time × group
		SHS		0.232±0.070		0.248±0.042		0.239±0.030		0.189±0.033	0.096±0.010	0.252±0.040		
Betaine	3.27	HS		1.120±0.308		1.191±0.302		1.158±0.154		1.017±0.054	0.814±0.186	2.176±0.379	0.0079	time × group
		SHS		0.838±0.072		0.826±0.089		0.843±0.093		1.316±0.599	0.549±0.056	1.033±0125		
Formate	8.45	HS		0.174±0.108		0.196±0.035		0.266±0.056		0.564±0.066	0.162±0.021	0.178±0.026	0.0001	time × group
		SHS		0.084±0.008		0.238±0.055		0.268±0.019		0.480±0.031	0.156±0.023	0.191±0.018		
Phenylalanine	7.31	HS		0.105±0.014		0.177±0.024		0.080±0.014		0.104±0.006	0.118±0.010	0.249±0.026	<0.0001	time × group
		SHS		0.094±0.009		0.127±0.009		0.073±0.008		0.062±0.005	0.109±0.003	0.162±0.009		
Pyruvate	2.36	HS		0.129±0.020		0.198±0.026		0.185±0.027		0.224±0.024	0.140±0.006	0.321±0.012	<0.0001	time × group
		SHS		0.127±0.018		0.195±0.026		0.108±0.010		0.141±0.013	0.156±0.018	0.257±0.031		
Creatinine	4.05	HS		0.093±0.033		0.092±0.017		0.094±0.019		0.109±0.021	0.059±0.015	0.251±0.021	0.0002	time × group
		SHS		0.167±0.029		0.215±0.056		0.154±0.019		0.114±0.044	0.096±0.021	0.197±0.025		
Valine	1.04	HS		0.316±0.046		0.446±0.031		0.295±0.055		0.278±0.034	0.341±0.021	0.396±0.033	0.0005	time × group
		SHS		0.332±0.034		0.167±0.029		0.206±0.019		0.233±0.013	0.309±0.028	0.283±0.013		
Creatine	3.93	HS		0.425±0.071		0.675±0.181		0.408±0.075		0.349±0.032	0.452±0.023	1.215±0.070	<0.0001	time group
		SHS		0.412±0.024		0.568±0.049		0.374±0.034		0.382±0.065	0.474±0.021	0.951±0.085		
Glutamate	2.34	HS		0.169±0.014		0.231±0.011		0.455±0.119		0.365±0.039	0.294±0.021	0.508±0.043	<0.0001	time × group
		SHS		0.210±0.029		0.259±0.028		0.359±0.024		0.281±0.024	0.279±0.024	0.350±0.026		
Acetoacetate	2.22	HS		0.560±0.089		1.086±0.177		0.870±0.212		1.633±0.419	1.030±0.166	1.994±0.254	0.0073	time × treatment
		SHS		0.723±0.151		1.753±0.419		1.838±0.177		3.942±0.637	1.110±0.138	3.320±0.752		
Cholines	3.21	HS		0.725±0.213		0.651±0.064		0.535±0.073		0.535±0.032	0.289±0.022	0.380±0.032	<0.0001	group
		SHS		0.834±0.085		0.894±0.096		0.829±0.060		0.745±0.053	0.313±0.011	0.391±0.023		
Glycine	3.55	HS		0.670±0.164		1.055±0.049		1.537±0.271		1.372±0.107	1.408±0.103	1.649±0.077	0.0021	group
		SHS		0.696±0.161		0.899±0.149		0.719±0.144		1.135±0.283	1.123±0.193	1.403±0.157		
Threonine	4.24	HS		0.475±0.114		0.542±0.082		0.329±0.051		0.319±0.050	0.399±0.070	0.658±0.085	0.0122	group
		SHS		0.603±0.089		0.662±0.095		0.497±0.065		0.325±0.136	0.469±0.047	0.512±0.051		
Isoleucine	1.01	HS		0.160±0.015		0.227±0.012		0.399±0.204		0.272±0.061	0.186±0.013	0.188±0.015	n/a	
		SHS		0.194±0.026		0.232±0.024		0.119±0.019		0.137±0.012	0.163±0.015	0.141±0.005		
Isobutyrate	1.17	HS		0.223±0.083		0.236±0.091		0.524±0.119		0.524±0.127	1.105±0.332	0.647±0.121	n/a	
		SHS		0.777±0.349		2.963±1.921		0.957±0.319		1.560±0.640	1.319±0.324	2.205±0.997		
Leucine	0.96	HS		0.390±0.047		0.509±0.029		0.778±0.346		0.569±0.108	0.377±0.022	0.433±0.032	n/a	
		SHS		0.437±0.057		0.489±0.039		0.330±0.045		0.331±0.022	0.349±0.028	0.317±0.012		
Lipophilic metabolites by ^1^H–NMR														
Total Triglycerides	0.87	HS		24.433±6.322		33.194±5.831		101.047±26.995		168.324±71.707	16.813±2.778	11.310±1.180	0.0004	group
		SHS		77.615±40.553		73.733±21.553		70.655±13.842		69.278±19.516	21.048±4.796	24.020±6.859		
Cholesterol	0.70	HS		3.156±0.775		4.144±0.719		10.115±2.288		13.042±5.152	2.383±0.412	1.716±0.212	0.0012	group
		SHS		6.500±2.259		7.594±1.950		8.118±1.6411		7.847±2.092	3.105±0.820	3.999±1.303		
PUFA	2.85	HS		6.482±1.914		8.055±1.092		12.278±2.417		17.120±7.362	7.840±1.403	5.217±0.658	n/a	
		SHS		13.126±5.265		15.178±3.400		10.684±2.931		10.937±2.931	9.460±2.440	12.453±4.016		
TAG	4.17	HS		2.889±0.811		3.858±0.753		14.737±4.901		27.610±12.470	1.446±0.283	1.044±0.158	n/a	
		SHS		12.421±7.879		10.259±3.442		9.348±1.859		9.166±2.675	1.773±0.411	2.120±0.601		
Phosphotidyl Ethanolamine	3.10	HS		1.590±0.0.523		1.396±0.209		3.004±0.710		3.205±1.611	1.503±0.294	1.106±0.490	n/a	
		SHS		2.209±1.093		2.968±0.778		2.173±0.750		2.090±0.372	1.531±0.646	1.908±0.513		
Cholines	3.23	HS		4.148±1.100		5.491±0.851		12.440±2.804		14.731±6.734	2.222±0.282	1.668±0.246	n/a	
		SHS		8.510±2.815		10.543±2.911		11.330±2.605		10.872±3.205	1.904±0.512	3.655±0.850		
UFA	5.35	HS		19.306±5.049		25.757±4.356		75.364±20.803		130.103±57.518	14.479±2.837	10.251±1.584	n/a	
		SHS		61.280±34.619		59.608±19.031		53.775±10.450		53.164±15.240	13.466±4.007	26.537±6.486		

Group was defined as EU-AGS vs. IBA-AGS vs. rat. Treatment indicated sham vs. hemorrhagic shock. For all lipophilic parameters, difference in group was IBA>rat. ANOVA analysis included sham surgery animals as a treatment. n = 5–7 for all groups. ANVOA value and significant differences (*p*<0.05, Tukey) are indicated. TAG, triacylglycerol; PUFA, Polyunsaturated fatty acids; UFA, unsaturated fatty acids.

**Figure 5:**
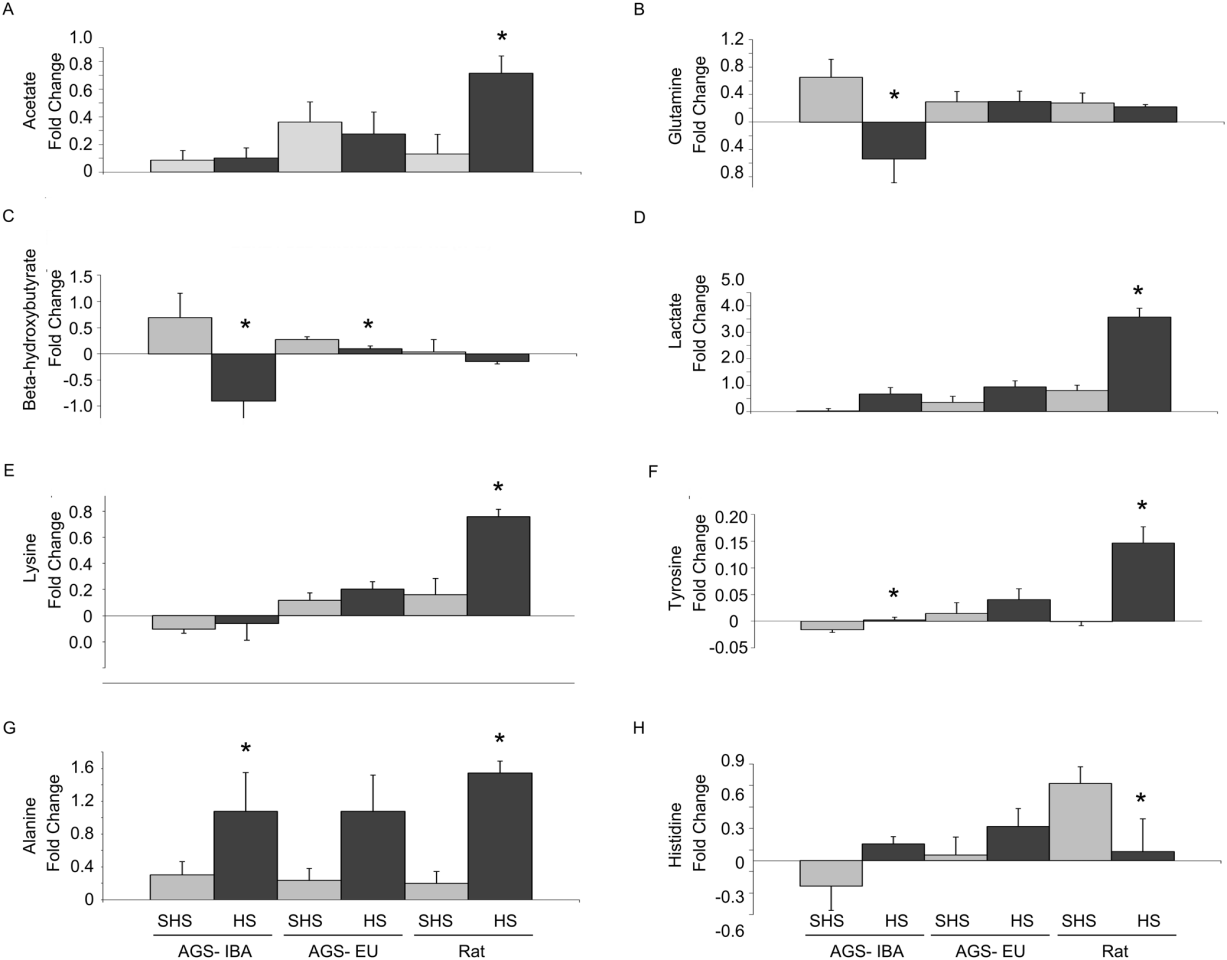
Fold change in plasma metabolites before and after hemorrhagic shock. Fold change in circulating metabolites before and after hemorrhagic shock (HS) or sham hemorrhagic shock (SHS) in winter interbout arousal (IBA) arctic ground squirrels, summer euthermic (EU) arctic ground squirrels, and rats. Data presented as mean ± SEM. Asterisks indicate p<0.05 vs. SHS of same group, Tukey test. n = 6–8 for all groups. Concentrations of metabolites are shown in Table 2.

Eighteen of these 25 metabolites had a significant concentration change between the start of HS and three hours post-HS. All of these showed a significant difference between rat, EU-AGS, and IBA-AGS (*p*<0.05, ANOVA, time × group and time × group × treatment; Table 2). Of these, only eight show a difference that was due to the HS (*p*<0.05, ANOVA, time × group × treatment; Table 2). These included acetate, alanine, glutamine, histidine, DBHB, lactate, lysine, and tyrosine. The fold change in these metabolites is shown in Figure 5. The I/R injury-prone rats had greater increases as compared to shams in acetate (*p* = 0.0069, 2-way ANOVA, time × treatment), lactate (*p*<0.0001, 2-way ANOVA, time × treatment), lysine (p = 0.0016), tyrosine (*p* = 0.0003, 2-way ANOVA, time × treatment), alanine (*p* = 0.0002, 2-way ANOVA, time × treatment), and less of an increase than sham in histidine (*p* = 0.0345, 2-way ANOVA, time × treatment). The greatest difference was seen in lactate which had a 3.6-fold increase after HS. In contrast, EU-AGS showed significant changes only in a decrease in DBHB (p = 0.0245, 2-way ANOVA, time × treatment), for these metabolites. IBA-AGS showed increases in tyrosine (*p* = 0.0007, 2-way ANOVA, time × treatment) and alanine (*p* = 0.0245, 2-way ANOVA, time × treatment), and decreases in glutamine (*p* = 0.0183, 2-way ANOVA, time × treatment) and DBHB (*p* = 0.0337, 2-way ANOVA, time × treatment).

## Discussion

This study shows for the first time that during global I/R the metabolic “fingerprint” of the I/R injury-prone rats shifts while those of the AGS remains unaltered. Specifically, rats show metabolic alterations during global I/R that correspond to an increase in gluconeogenesis, glycolysis and anaerobic metabolism in addition to markers for organ dysfunction. In contrast, EU-AGS maintain stable concentrations of lipophilic and hydrophilic metabolites while IBA-AGS show increases in only two of the metabolites measured, tyrosine and alanine, and a decrease in glutamine and DBHB during HS.

### Species’ metabolic differences before I/R

The degree of susceptibility to I/R injury may lie in baseline metabolic differences between I/R injury prone species and those that have innate resistance. Although the naïve rats had differing overall metabolic profiles than the naive EU- or IBA-AGS, the quantities of metabolites only differed for four metabolites (acetate, choline, glutamine, and threonine). Interestingly, only two of those, acetate and glutamine, also had significant changes due to HS that differed between the rats and AGS. Acetate [33], [34] and glutamate [35], [36] have been shown to have beneficial effects in rat models of HS. Naïve rats had higher concentrations than AGS in both acetate and glutamate but still had I/R damage after HS. That the rats had I/R damage even though they had higher baseline levels of these protective metabolites, suggests that it is not a variation in metabolic process under neutral conditions that enable the AGS to tolerate I/R, but that protection may stem from metabolic process during I/R that prevent injury.

### Metabolic instability during HS in the I/R injury prone species

In rats, a species vulnerable to I/R injury [11] there were district changes in circulating metabolites due to HS. Increased plasma lactate and alanine, two gluconeogenic precursors, during HS indicate active carbohydrate and anaerobic metabolism [37]. These results are consistent with previous studies in our lab that showed that glucose increased in the rat during I/R and for the three hours following reperfusion [11]. Lactate is a well-established herald of poor prognosis after I/R injury [38], [39]. It is one of four hydrophilic metabolites (glucose, glutamate, lactate, and DBHB) that have been found by other ^1^H–NMR metabolomic studies on I/R to have a high correlation with morbidity [40], [41]. Alanine is also an indicator of dysfunctional lipid metabolism. This study extends these prior studies by showing that these biomarkers of poor prognosis are absent in an I/R injury-resistant species.

### Metabolic homeostasis during I/R in hibernators

Another purpose of this study was to identify changes in circulating metabolites and metabolic profiles that would be indicative of metabolic pathways in the hibernators that might contribute to their innate protective mechanism against I/R injury, systemic inflammation, and multi organ damage. AGS response to I/R was assessed in their summer active season (EU) and during their winter hibernation seasons (IBA) as primary fuel sources switch from a glucose- to lipid-based metabolism, respectively. Despite the seasonal switch in fuel sources in AGS, we have shown that both the IBA- and EU-AGS resist I/R injury (5). Here we demonstrate that the mechanism behind the response is not dependent on seasonal differences in their metabolome but was associated with overall homeostasis. Independence from anaerobic metabolism, indicated by a lack of lactate accumulation also contributed to overall homeostasis. Although differences were noted between EU- and IBA-AGS, the overall fingerprints for both groups were undisturbed by I/R.

Interestingly, the EU-AGS and IBA-AGS data cluster individually, with barely any overlap in naïve animals, but this clustering is lost after surgery (SHS or HS; Fig. 1, Fig. S12). Thus while AGS resist loss of metabolic homeostasis after HS, they do not resist metabolic disturbance caused by surgery alone. This observation emphasizes that the AGS resistance is specific to I/R and not to trauma in general.

Changes in specific metabolites are consistent with low demand for glucose, gluconeogenesis and transport of substrates from peripheral sources to the liver. For example, circulating glutamine, a gluconeogenic precursor, decreased in IBA-AGS during I/R, and the lack of an I/R-induced increase in lactate in AGS of either season indicates that anaerobic metabolism was not utilized in the AGS during I/R. This agrees with previous work showing that an avoidance of lactate build up is a key feature of ischemic preconditioning and that lactate does not accumulate in other models of I/R during the winter season in AGS and other species of hibernating ground squirrels [11], [13], [27], [42]–[44]. Similarly, the decreases in the fold change of circulating levels of DBHB during hemorrhage in both AGS groups concurs with a low demand for glucose noted previously in IBA and hibernating ground squirrels. Metabolomic fingerprints of IBA- and EU-AGS were consistent with reports from other species showing a metabolic switch during the hibernation season [5]–[7], [14], [15], [45]–[48]. Surprisingly, both groups of animals avoided anaerobic glucose metabolism during I/R, consistent with resistance from I/R injury. DBHB is protective during I/R [49]–[52], presumably from the ketone bodies direct entrance into the TCA cycle and the avoidance of the conversion of pyruvate to lactate and development of acidosis [53]–[55]. Similar metabolomes in both groups of AGS suggest that metabolic flexibility persists in AGS during the summer season.

The seasonal shift in metabolism is consistent with other studies in other species of ground squirrels; however, the specific metabolites differed from these other studies. The seasonal differences in hydrophilic metabolites between summer and winter AGS reported here vary from those previously reported from ^1^H–NMR analysis. Serkova et al. found that lactate and alanine concentrations were higher in summer versus IBA ground squirrels [13]. However, that study was focused on monitoring metabolic changes in the liver during hibernation cycles in ground squirrels. The differences noted could be due to the tissue examined (liver versus plasma). It may also be due to the fact that the IBA animals used in the current study were completely euthermic and showed no signs (e.g. reduced body temperature) of entering into a torpor bout. In contrast, Serkova and colleagues specifically used IBA animals that dropped core body temperature and were beginning to enter torpor. Previous studies have shown proteomic and metabolomic differences between normothermic IBA animals and those who are in various stages of torpor [19]–[21]. In this study, we used normothermic IBA animals, which had been handled gently ∼20 hours prior to the start of the experiment to induce arousal. This was done for two reasons: 1) to examine endogenous I/R protective mechanisms outside those of reduced body temperature, and 2) to avoid confounding results stemming from torpid animals that have vastly depressed cardiovascular, respiratory, and metabolic rates [56], [57].

### Lipid metabolome remains unaltered during hemorrhagic shock

With regard to lipophilic metabolites, we did not see significant differences between summer and winter season ground squirrels noted by others [13], [16], likely due to differences in study design as discussed above. However, there was a difference between the two species with rats having lower levels of lipophilic metabolites than either AGS in either season overall. Others have demonstrated that products of lipid metabolism are protective during I/R [51], [52], [58], [59]. As there was no significant change in any of the lipophilic metabolites from before to after HS within a group (rats, IBA-AGS, and EU-AGS), it is unlikely that these contributed to protective biochemical mechanisms in the AGS or injurious mechanisms in the rat. There is a well-documented difference in lipid metabolism between summer euthermic and winter ground squirrels [6], [13]–[22], [60]. With ^1^H–NMR analysis of lipophilic metabolites, Serkova et al. found that only polyunsaturated fatty acids (PUFA) levels were greater in IBA versus summer animals. However, most of the differences in the lipophilic metabolites were between summer and torpid animals [13]. Epperson et al. found that cholesterol levels in plasma were higher in IBA versus summer animals [16]. In the current study, the IBA animals tended to have higher cholesterol levels, but the difference was not significant. These discrepancies are most likely due to high variation between individuals in each group due to differences in IBA duration and how far along in the process each animal was. In both of the previous studies, IBA animals were studied at different time points within the IBA process (entrance into torpor and 3 hours after euthermic body temperature was reached), in a synchronized fashion. During each IBA, the animal goes through many dynamic physiological and biochemical alterations that are specific to the arousal/torpor cycle [16], [19], [21]. The present study sought to examine metabolic mechanisms that differ between the summer and winter season, versus between the IBA and torpid state. As such, IBAs were not synchronized for experiments which might contribute to the higher variation in metabolite concentrations in this group.

### Organ damage markers

In addition to flagging alterations in metabolic pathways and preferential fuel sources, increases in several of the metabolites are established as biomarkers for organ damage or dysfunction [27], [40], [43], [61], [62]. The I/R injury prone rats had increases in lactate, tyrosine, lysine, and acetate, markers of hepatic injury (Table 3; Fig. 5). Lactate, a sign for anaerobic metabolism is also a marker of liver dysfunction and decreased ability of the liver to clear circulating excess lactate from the blood stream [63]. The same is true of tyrosine, lysine, and acetate. Increases in lactate and amino acids, such as tyrosine and lysine, are indicative of decreased hepatic function [61] as is an increase in acetate, one of the major endpoints in several hepatic metabolic pathways [43]. These results agree with previous studies in our lab that have shown from standard blood chemistry analysis that after HS, rats have increased serum markers for liver damage (aspartate aminotransferase; AST, and alanine aminotransferase; ALT [11]). Meanwhile, EU-AGS did not have an increase in any of the markers for organ damage after HS. Although IBA-AGS showed an increase in tyrosine, a marker for liver dysfunction, the increase was much less than observed in rats, consistent with absence of organ damage noted previously [11].

**Table 3:** ^1^H–NMR plasma biomarkers predictive of poor prognosis after hemorrhagic shock-induced global ischemia/reperfusion injury.

Anaerobic Metabolism	Carbohydrate Turnover	Liver Dysfunction
↑Lactate	↑Lactate	↑Lactate
	↑Acetate	↑Acetate
		↑Tyrosine
		↑Lysine

Previous studies have shown a difference in I/R tolerance between summer- and winter-season ground squirrels [5]–[7], [64]. These experiments have been performed on various isolated tissue preparations and have demonstrated resistance to I/R injury in winter, but not summer animals for liver, kidney, and small intestine. In contrast, our previous report detailed that both IBA- and EU-AGS were resistant to systemic inflammation and multi organ damage after global I/R [11]. Similarly, the brains of spring/summer euthermic ground squirrels have been shown to be resistant to I/R injury [10], [65]. As NMR is a relatively insensitive method that requires ∼2–4 mM of metabolites for detection, some metabolic perturbations may require more than three hours to reach detectable concentrations [41]. For example, allatoin, a marker for renal damage, has been found only to be distinguishable in the blood 24 hours after I/R [27]. In this study, allantoin levels differed between the treatment groups, but were not different between sham and hemorrhaged animals, perhaps pointing to some low-level renal dysfunction that was associated with the surgical procedure itself.

For metabolomic analysis, plasma is considered the biofluid of choice [41] and is well established for the evaluation of the metabolic status of various organs [40]. In principle, it represents an “average” physiological biochemical status of the organism as it interacts with all of the various tissues and organs. However, hibernating animals have the ability to regulate blood flow away from peripheral regions during hibernation and arousal [66]–[69]. Also, given the complexity of the hemodynamics during hemorrhage, there may be differential perfusion to various organs over the time course. Both of these factors may have affected the levels of metabolites that specific organs contributed to the plasma that was analyzed. In addition, EDTA was used as an anticoagulant. EDTA produces NMR peaks that can obscure detection of certain metabolites [70]. However, most of these metabolites can be quantified based on peaks in regions clear of the anticoagulant peaks [71].

## Conclusion

Systemic I/R injury, such as occurs in HS, is a leading cause of death in trauma patients [40]. Lack of oxygen and nutrients in conjunction with buildup of waste products during the ischemic period, followed by the inrush of oxygen and the generation of reactive oxygen species (ROS) during reperfusion induces metabolic alterations at the cellular level. By analyzing the metabolites and metabolic profile of a species known to be prone to I/R damage, both before and after I/R insult, we were able to establish biomarkers of metabolic perturbations and organ damage that precede HS-induced fatality. By then comparing the metabolic profile of the I/R-resistant AGS to these biomarkers, we discovered that, unlike the I/R injury prone rats, which experience distinct alterations in their metabolome during I/R, AGS are able to sustain their lipophilic and hydrophilic metabolic profiles. Specifically, AGS resist changes to metabolites involved in carbohydrate turnover, anaerobic metabolism, and organ dysfunction. This information suggests that the underlying factors involved in I/R resistance in these animals is not based in the seasonal switch in energy source from carbohydrate in the summer to lipid in the winter but in the maintenance of metabolic homeostasis. The stability of their metabolome during the period of limited nutrient and oxygen availability indicates that they may have better metabolic regulatory and/or compensatory mechanisms not utilized by homothermic species. These metabolic mechanisms could provide potential therapeutic interventions to alleviate the metabolic imbalances and subsequent inflammatory response and organ damage associated with global I/R.

## Supporting Information

Figure S1 **Experimental protocol for hemorrhagic shock.** Core body temperature and head temperature were maintained between 36.5 and 37.5°C with a warm water blanket under the animal and heat lamps above the animal from the start of surgical preparation until the end of post HS monitoring. Arrows indicate blood sampling timepoints. Hemorrhagic shock (HS); mean arterial pressure (MAP).
(TIF)

Figure S2 **H-C-HMQC verifying the identity of Acetate at 1.91 ppm, from extracted naïve rat sample to aid in peak identification.**
(TIF)

Figure S3 **^1^H–CPMG of unprocessed rat plasma.**
(TIF)

Figure S4 **H-H-COSY of hydrophilic fraction of extracted naive rat sample to aid in peak identification.**
(TIF)

Figure S5 **H-H-COSY of hydrophilic fraction of extracted naive rat sample to aid in peak identification.**
(TIF)

Figure S6 **H-C-HMQC of hydrophilic fraction of extracted naive rat sample to aid in peak identification.**
(TIF)

Figure S7 **H-C-HMQC of hydrophilic fraction of extracted naive rat sample to aid in peak identification.**
(TIF)

Figure S8 **H-C-HMQC of hydrophilic fraction of extracted naive rat sample to aid in peak identification.**
(TIF)

Figure S9 **^1^H–CPMG of unprocessed AGS plasma to aid in peak identification.**
(TIF)

Figure S10 **H-C-HSQC from unprocessed AGS plasma to aid in peak identification.**
(TIF)

Figure S11 **Example Jres NMR spectrum from unprocessed AGS plasma.**
(TIF)

Figure S12 **Hydrophilic metabolic profiles for naïve, before SHS, after SHS, before HS, and after HS rats (A; R^2^ = 0.75, Q^2^ = 0.15, permutation p = 0.90) summer euthermic AGS (B; R^2^ = 0.60, Q^2^ = 0.07, permutation p = 0.03) and winter IBA-AGS (C; R^2^ = 0.90, Q^2^ = 0.30, permutation p = 0.38).**
(TIF)

Table S1 **Experimental groups HS experiments.**
(DOCX)

Table S2 **Characteristics of AGS undergoing HS during the winter (IBA) season.**
(DOCX)

Table S3 **Characteristics of AGS undergoing HS during the summer (euthermic) season.**
(DOCX)

Table S4 **Characteristics of AGS undergoing SHS during the winter (IBA) season.**
(DOCX)

Table S5 **Characteristics of AGS undergoing SHS during the summer (euthermic) season.**
(DOCX)
