# Supplementary figures and images for: 1H–NMR Metabolomic Biomarkers of Poor Outcome after Hemorrhagic Shock are Absent in Hibernators
Source: PLoS One. 2014 Sep 11;9(9):e107493. doi: 10.1371/journal.pone.0107493 (PMC4161479; doi:10.1371/journal.pone.0107493)

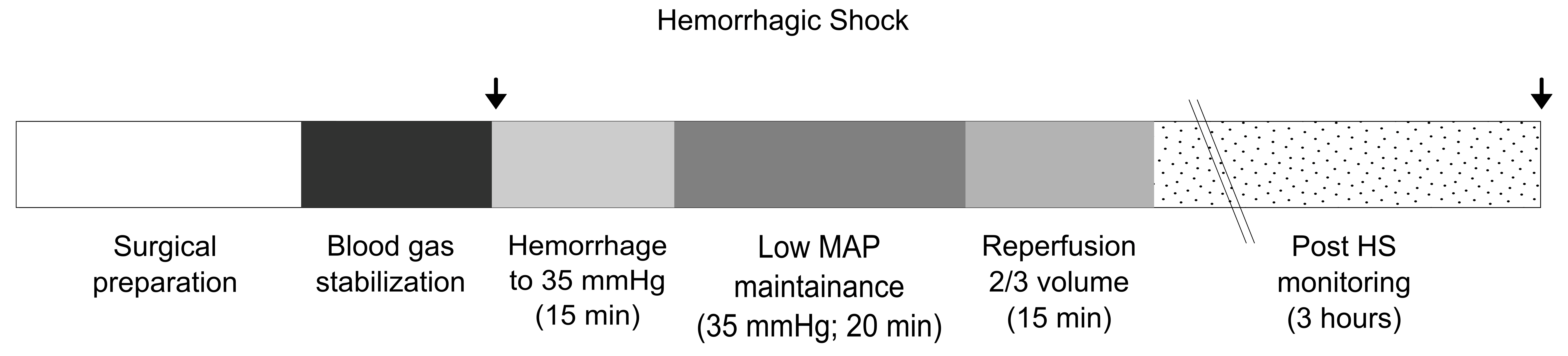

Supplement: Figure S1 — Experimental protocol for hemorrhagic shock. Core body temperature and head temperature were maintained between 36.5 and 37.5°C with a warm water blanket under the animal and heat lamps above the animal from the start of surgical preparation until the end of post HS monitoring. Arrows indicate blood sampling timepoints. Hemorrhagic shock (HS); mean arterial pressure (MAP). (TIF) [file pone.0107493.s001.tif]

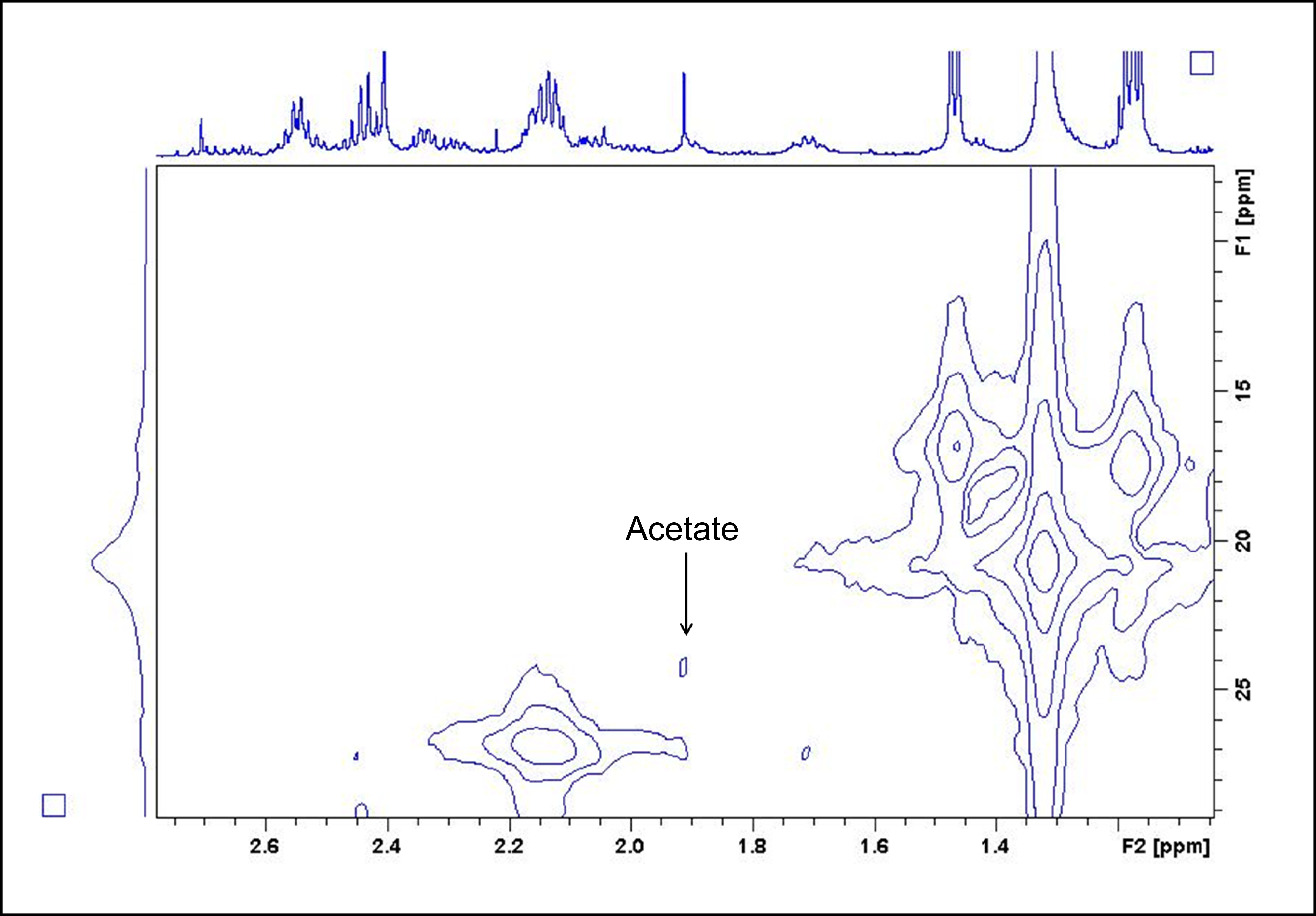

Supplement: Figure S2 — H-C-HMQC verifying the identity of Acetate at 1.91 ppm, from extracted naïve rat sample to aid in peak identification. (TIF) [file pone.0107493.s002.tif]

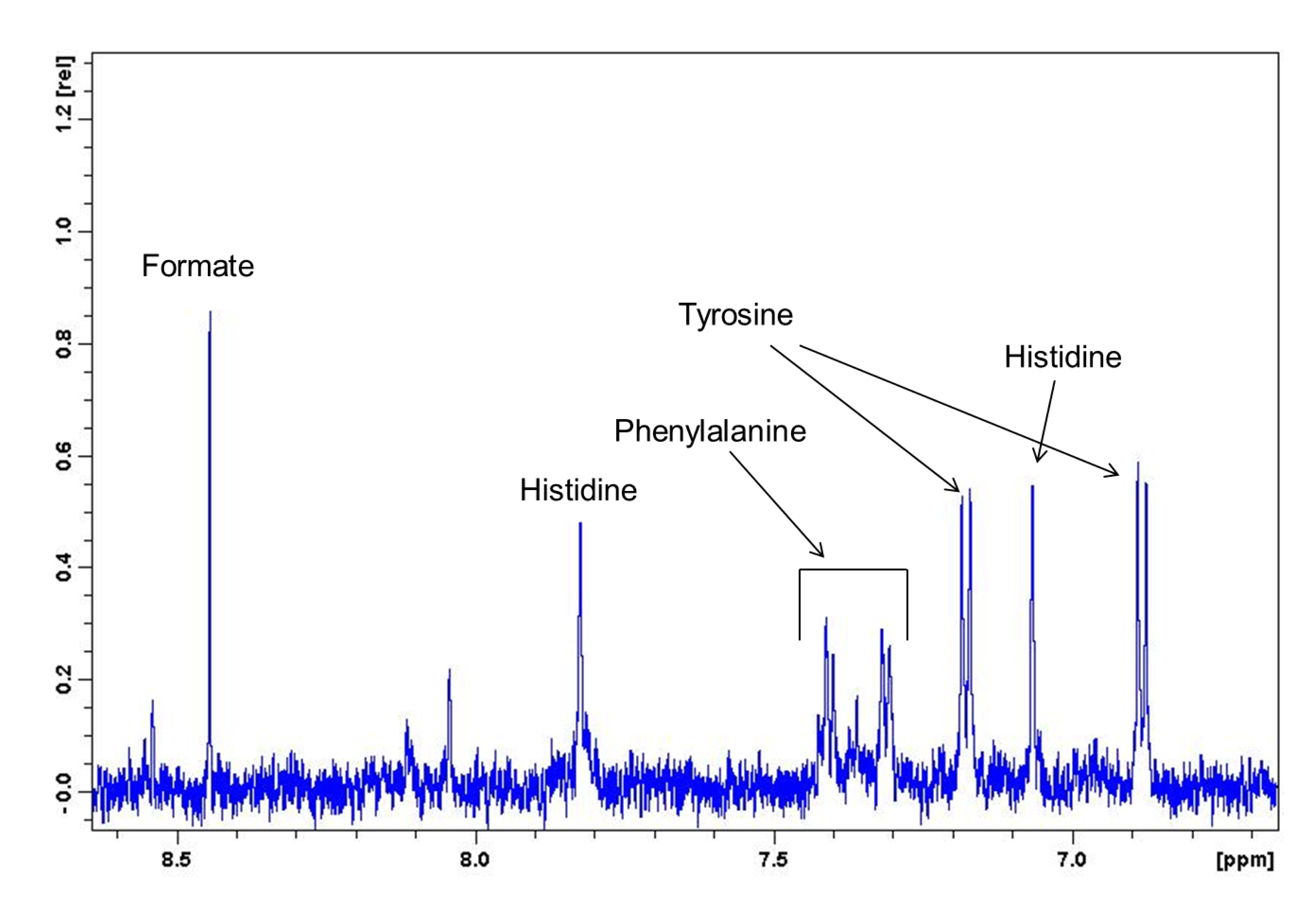

Supplement: Figure S3 — 1H–CPMG of unprocessed rat plasma. (TIF) [file pone.0107493.s003.tif]

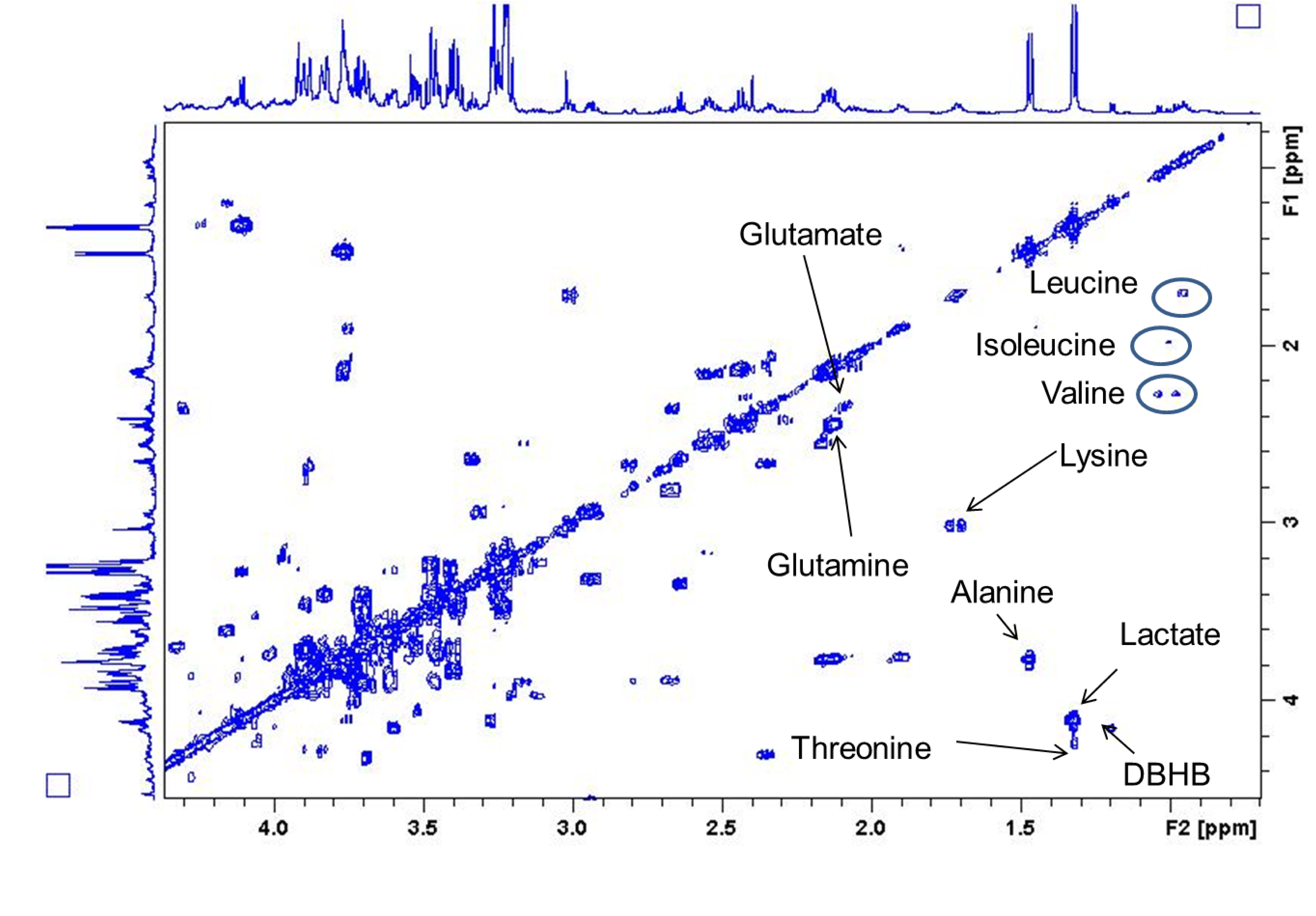

Supplement: Figure S4 — H-H-COSY of hydrophilic fraction of extracted naive rat sample to aid in peak identification. (TIF) [file pone.0107493.s004.tif]

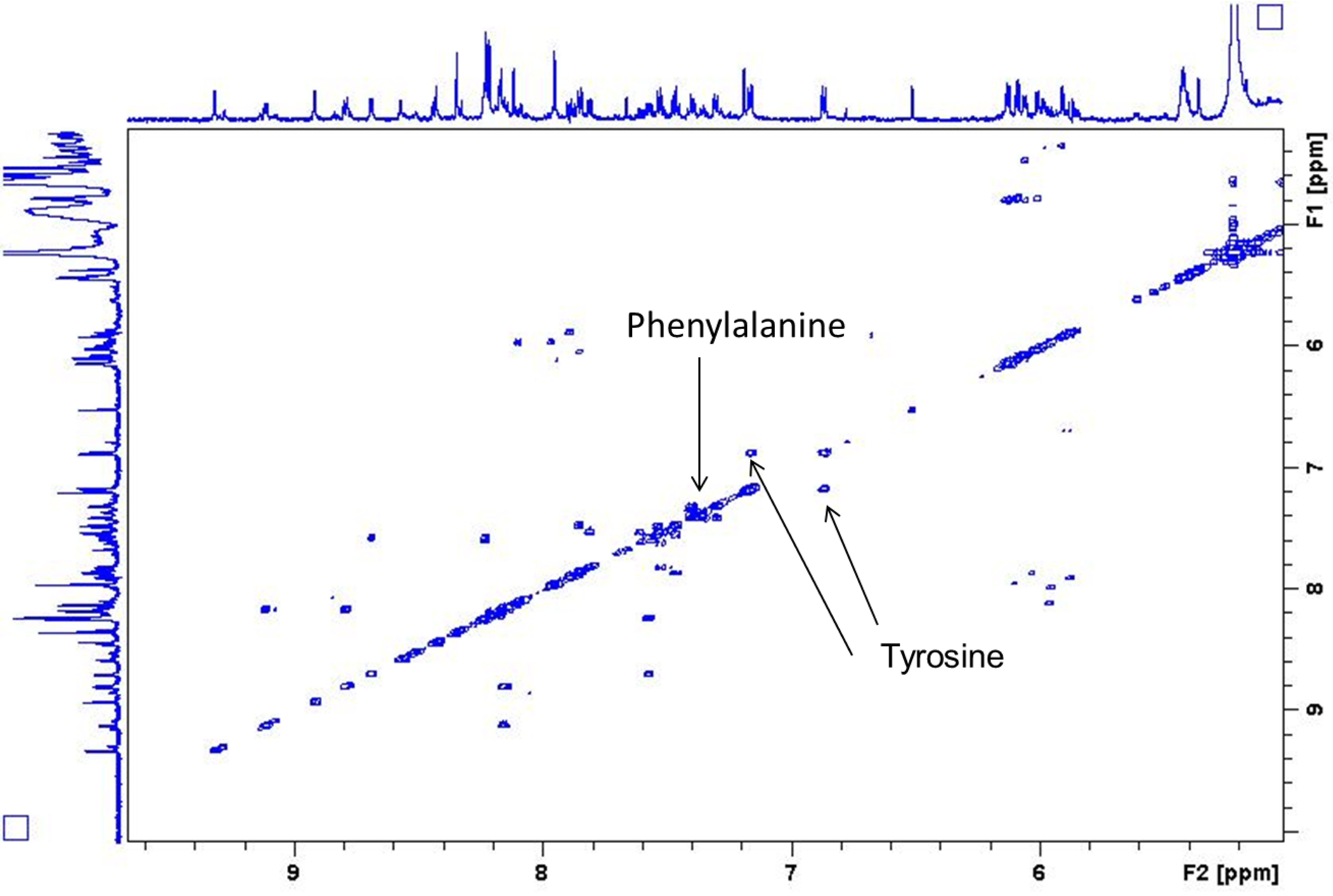

Supplement: Figure S5 — H-H-COSY of hydrophilic fraction of extracted naive rat sample to aid in peak identification. (TIF) [file pone.0107493.s005.tif]

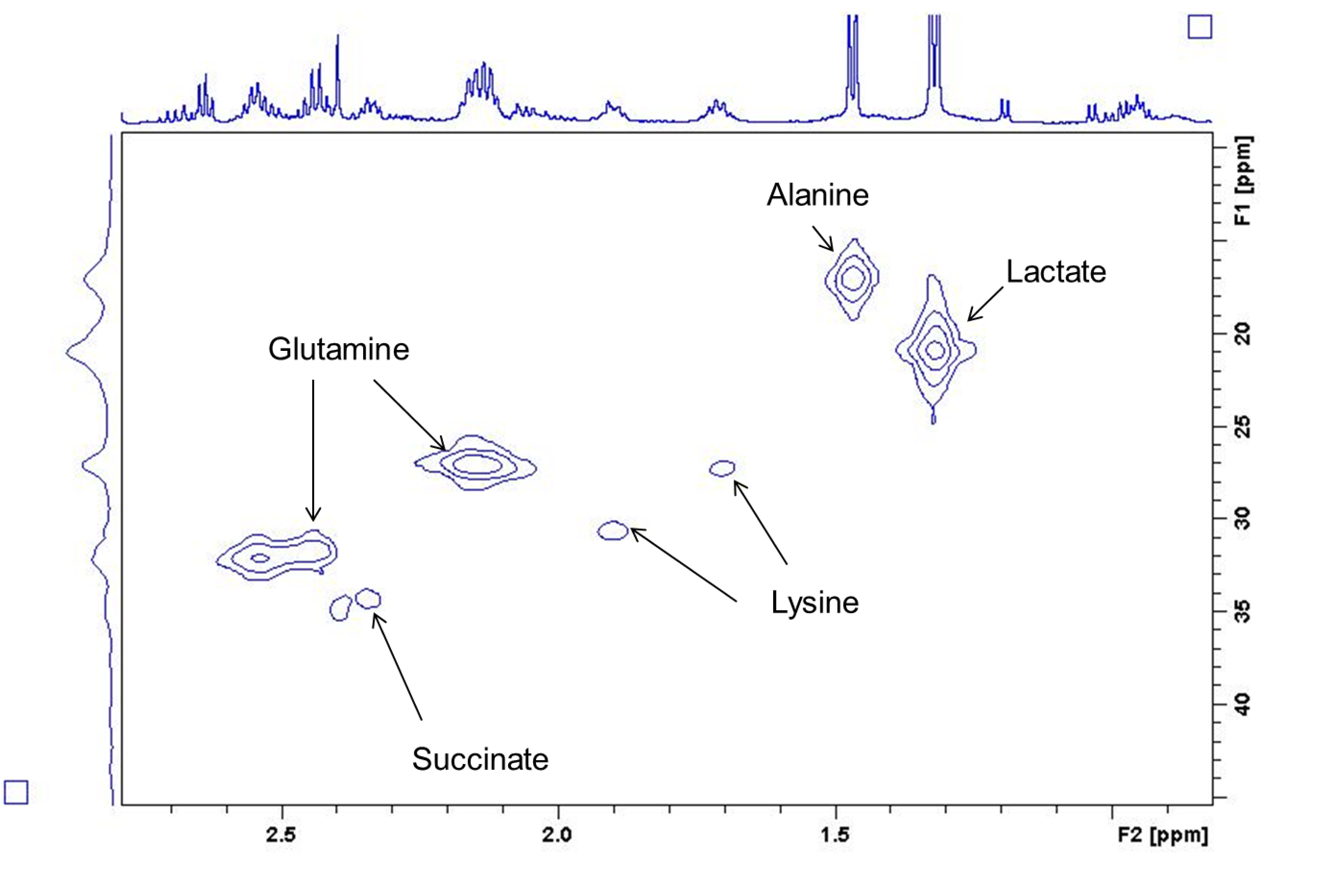

Supplement: Figure S6 — H-C-HMQC of hydrophilic fraction of extracted naive rat sample to aid in peak identification. (TIF) [file pone.0107493.s006.tif]

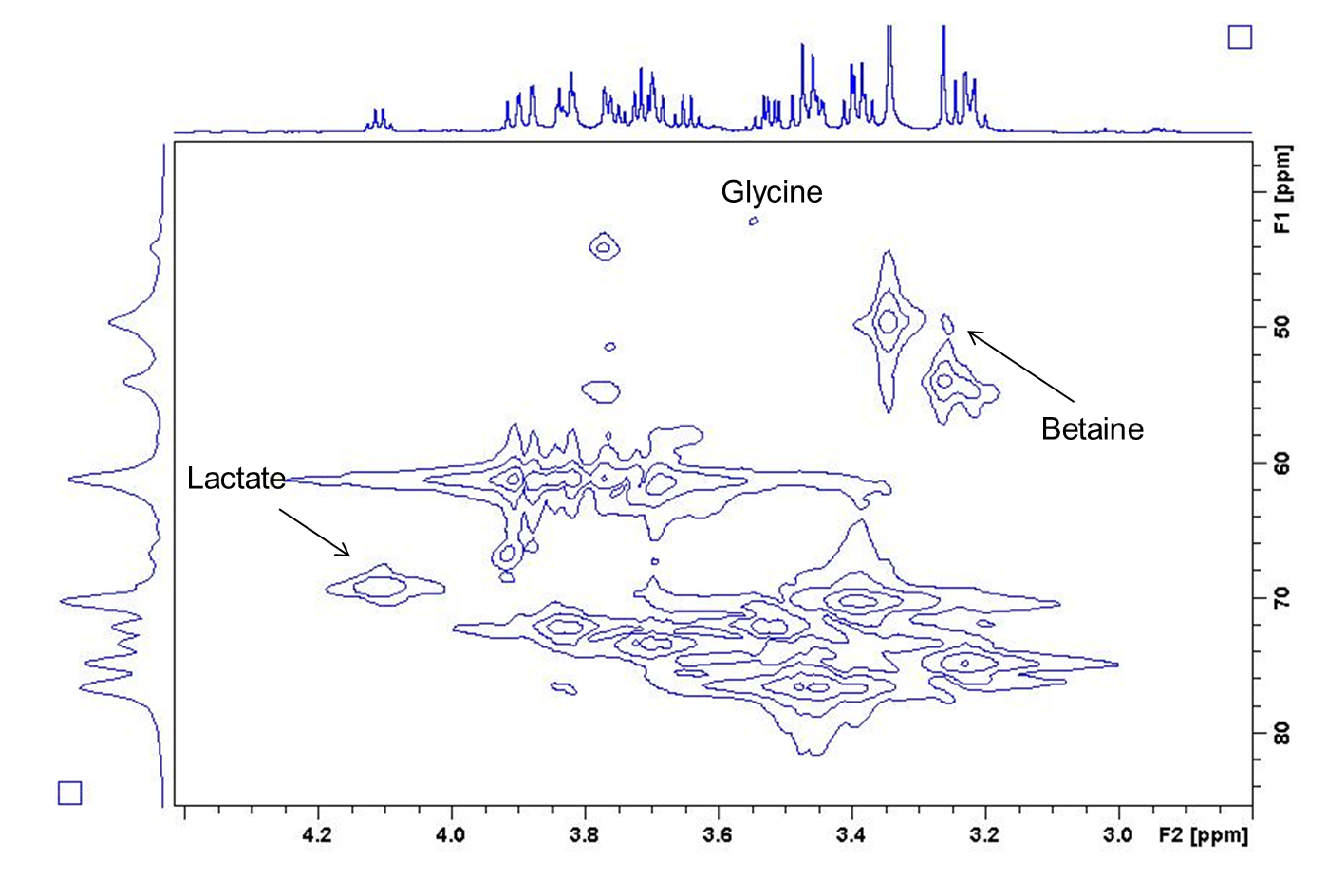

Supplement: Figure S7 — H-C-HMQC of hydrophilic fraction of extracted naive rat sample to aid in peak identification. (TIF) [file pone.0107493.s007.tif]

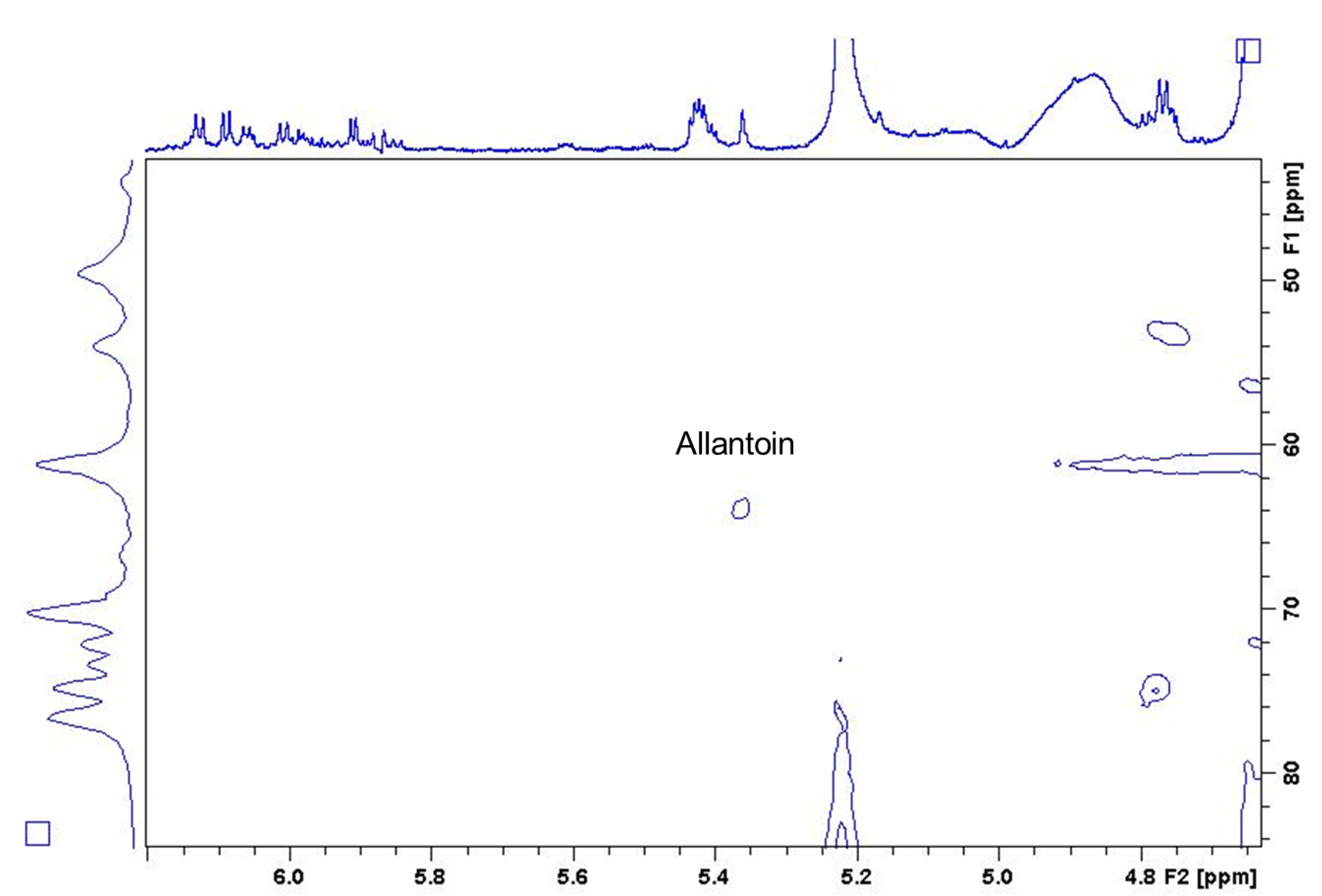

Supplement: Figure S8 — H-C-HMQC of hydrophilic fraction of extracted naive rat sample to aid in peak identification. (TIF) [file pone.0107493.s008.tif]

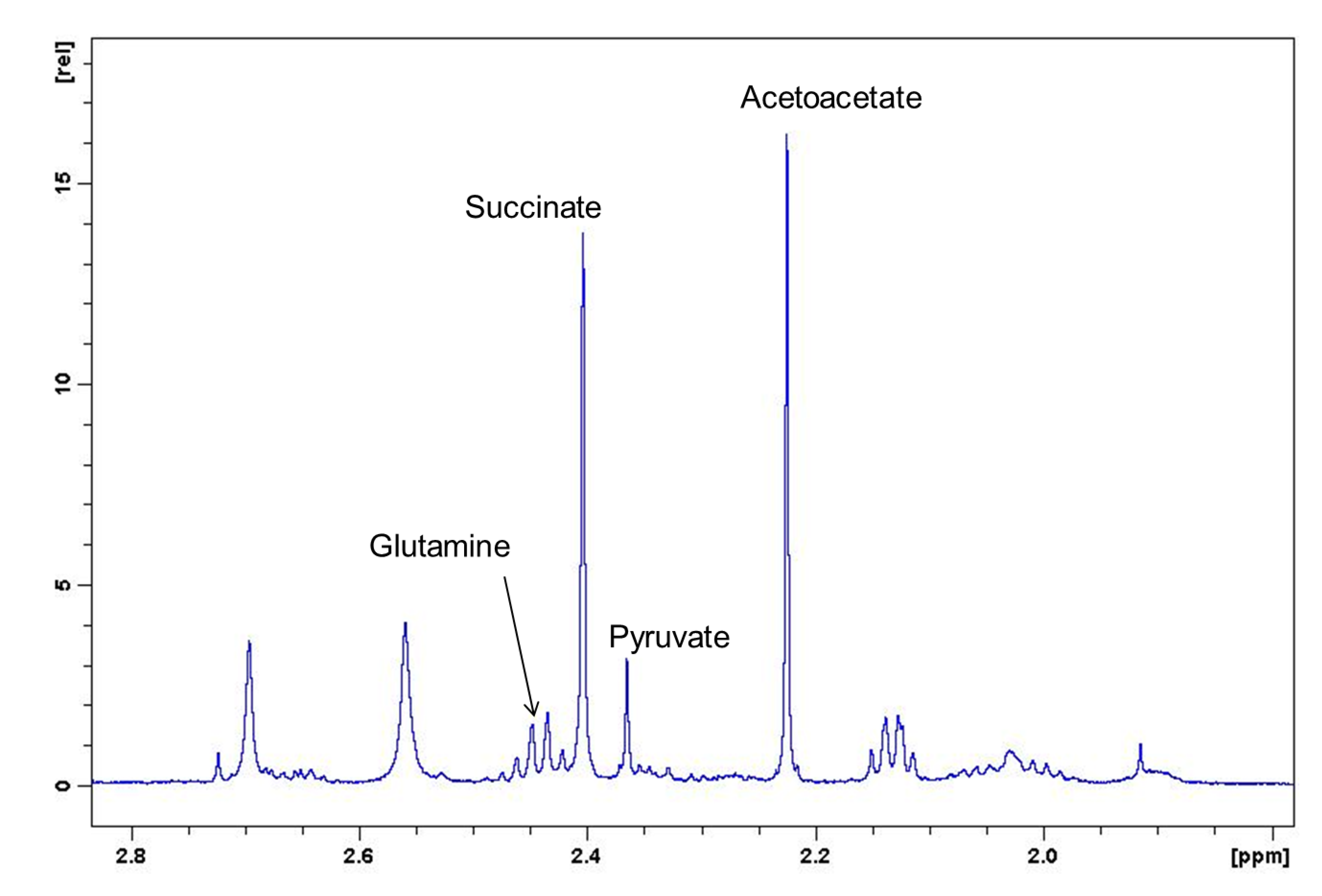

Supplement: Figure S9 — 1H–CPMG of unprocessed AGS plasma to aid in peak identification. (TIF) [file pone.0107493.s009.tif]

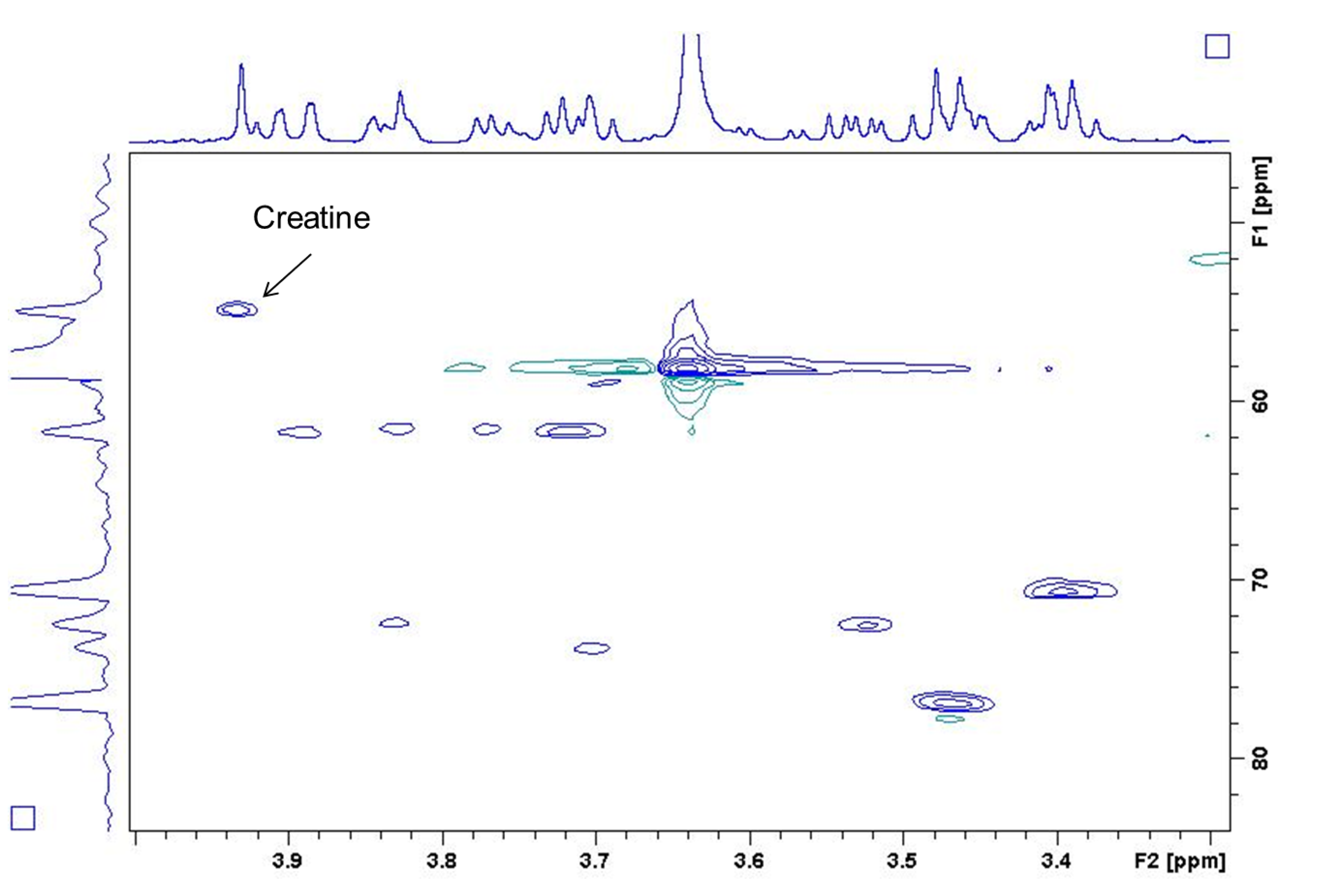

Supplement: Figure S10 — H-C-HSQC from unprocessed AGS plasma to aid in peak identification. (TIF) [file pone.0107493.s010.tif]

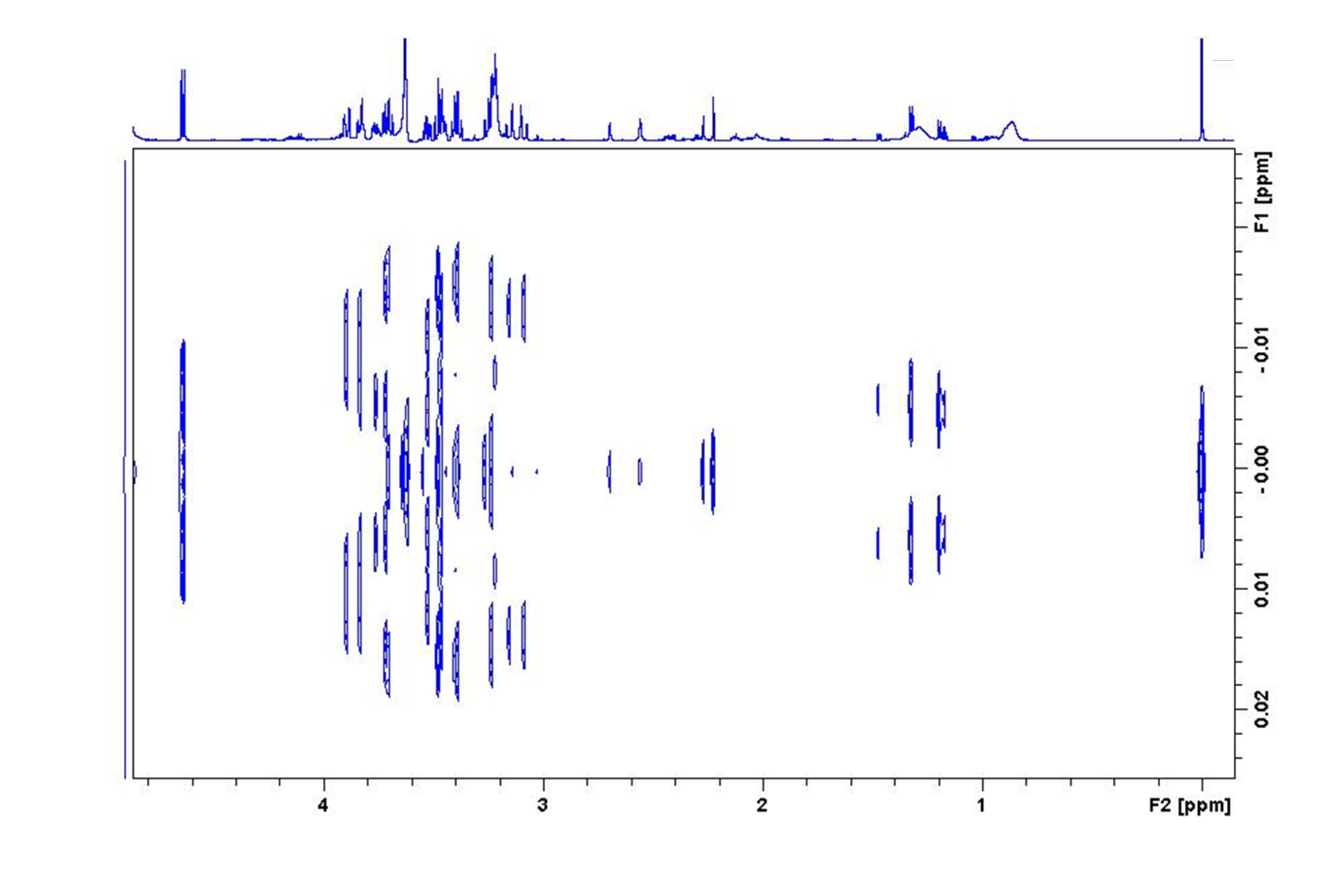

Supplement: Figure S11 — Example Jres NMR spectrum from unprocessed AGS plasma. (TIF) [file pone.0107493.s011.tif]
