# Supplementary material for: 1H–NMR Metabolomic Biomarkers of Poor Outcome after Hemorrhagic Shock are Absent in Hibernators
Source: PLoS One. 2014 Sep 11;9(9):e107493. doi: 10.1371/journal.pone.0107493 (PMC4161479; doi:10.1371/journal.pone.0107493)
Supplement: Table S1 — Experimental groups HS experiments. (DOCX) [file pone.0107493.s013.docx]

Table S1. Experimental groups HS experiments

| **Experiment** | **Group** | **Treatment** | **n**  **available** | **n**  **per parameter** | **n**  **died*** |
| --- | --- | --- | --- | --- | --- |
| HS | Rat | naive | 8 | 8 | 0 |
|  |  | HS | 6 | 6 | 0 |
|  |  | SHS | 7 | 7 | 0 |
|  | AGS-EU | naive | 7 | 7 | 0 |
|  |  | HS | 6 | 6 | 0 |
|  |  | SHS | 7 | 7 | 0 |
|  | AGS-IBA | naive | 7 | 7 | 0 |
|  |  | HS | 6 | 6 | 0 |
|  |  | SHS | 8 | 7 | 0 |

HS: Hemorrhagic shock, SHS: sham hemorrhagic shock, AGS: arctic ground squirrel, EU: euthermic (summer), IBA: interbout arousal (winter). Animals were euthanized under anesthesia at the end of the protocol.
