# Supplementary material for: 1H–NMR Metabolomic Biomarkers of Poor Outcome after Hemorrhagic Shock are Absent in Hibernators
Source: PLoS One. 2014 Sep 11;9(9):e107493. doi: 10.1371/journal.pone.0107493 (PMC4161479; doi:10.1371/journal.pone.0107493)
Supplement: Table S2 — Characteristics of AGS undergoing HS during the winter (IBA) season. (DOCX) [file pone.0107493.s014.docx]

**Table S2. Characteristics of AGS undergoing HS during the winter (IBA) season.**

| Animal number | 09-56 | 08-83 | 08-40 | 08-68 | 10-05 | 10-48 |
| --- | --- | --- | --- | --- | --- | --- |
| Season | Winter | | | | | |
| Age | Adult | Adult | Adult | Adult | Adult | Adult |
| Sex | Female | Female | Female | Male | Female | Female |
| Mass (g) | 862 | 1123 | 1044 | 1056 | 559 | 721 |
| First day of spontaneous torpor | 18-Aug-10 | 18-Aug-10 | 17-Aug-10 | 4-Aug-10 | 29-Aug-10 | 2-Oct-10 |
| Experiment day | 7-Jan-11 | 11-Jan-11 | 24-Jan-11 | 27-Jan-11 | 17-Feb-11 | 18-Feb-11 |
| No. of spontaneous torpor bouts prior to HS | 11 | 16 | 14 | 14 | 13 | 13 |
| Ave length of previous 3 torpor bouts (days) | 15 | 11 | 11 | 14 | 18 | 18 |
| Day in bout | 6 | 4 | 4 | 4 | 2 | 11 |
| Tb at induced arousal (°C) | 3 | 3.3 | 2.9 | 3 | 4 | 4.7 |
| Blood volume removed (% total) | 16 | 15 | 22 | 24 | 28 | 37 |
