# Supplementary material for: 1H–NMR Metabolomic Biomarkers of Poor Outcome after Hemorrhagic Shock are Absent in Hibernators
Source: PLoS One. 2014 Sep 11;9(9):e107493. doi: 10.1371/journal.pone.0107493 (PMC4161479; doi:10.1371/journal.pone.0107493)
Supplement: Table S4 — Characteristics of AGS undergoing SHS during the winter (IBA) season. (DOCX) [file pone.0107493.s016.docx]

**Table S4. Characteristics of AGS undergoing SHS during the winter (IBA) season.**

| Animal number | 08-51 | 09-86 | 08-97 | 10-09 | 10-24 | 10-28 | 10-33 | 10-12 |
| --- | --- | --- | --- | --- | --- | --- | --- | --- |
| Season | Winter | | | | | | | |
| Age | Adult | Adult | Adult | Adult | Adult | Adult | Adult | Adult |
| Sex | Female | Male | Female | Female | Male | Male | Male | Female |
| Mass (g) | 671 | 704 | 904 | 454 | 738 | 729 | 731 | 438 |
| First day of spontaneous torpor | 7-Aug-10 | 18-Aug-10 | 17-Aug-10 | 29-Aug-10 | 7-Dec-10 | 16-Oct-10 | 1-Nov-10 | 16-Sep-10 |
| Experiment day | 21-Jan-11 | 18-Jan-11 | 19-Jan-11 | 28-Jan-11 | 3-Feb-11 | 4-Feb-11 | 11-Feb-11 | 23-Feb-11 |
| No. of spontaneous torpor bouts prior to SHS | 12 | 13 | 11 | 12 | 5 | 10 | 8 | 11 |
| Ave length of previous 3 torpor bouts (days) | 10 | 14 | 18 | 14 | 14 | 17 | 14 | 14 |
| Day in bout | 3 | 5 | 13 | 8 | 7 | 6 | 7 | 5 |
| Tb at induced arousal (°C) | 3.1 | not taken | 3.3 | 4 | 3.7 | 4.2 | 3.8 | 3.7 |
