# Supplementary material for: 1H–NMR Metabolomic Biomarkers of Poor Outcome after Hemorrhagic Shock are Absent in Hibernators
Source: PLoS One. 2014 Sep 11;9(9):e107493. doi: 10.1371/journal.pone.0107493 (PMC4161479; doi:10.1371/journal.pone.0107493)
Supplement: Table S5 — Characteristics of AGS undergoing SHS during the summer (euthermic) season. (DOCX) [file pone.0107493.s017.docx]

**Table S5. Characteristics of AGS undergoing SHS during the summer (euthermic) season.**

| Animal number | 09-76 | 09-83 | 10-16 | 10-27 | 10-14 | 09-68 | 10-30 |
| --- | --- | --- | --- | --- | --- | --- | --- |
| Season | Summer | | | | | | |
| Age | Adult | Adult | Adult | Adult | Adult | Adult | Adult |
| Sex | Female | Male | Male | Male | Female | Female | Female |
| Mass (g) | 1036 | 554 | 464 | 658 | 617 | 833 | 446 |
| Last day of torpor during previous season | 10-Nov-10 | 10-Feb-11 | 20-Feb-11 | 09-Feb-11 | 28-Feb-11 | 5-Jan-11 | 27-Feb-11 |
| Experiment day | 7-Jun-11 | 10-Jun-11 | 16-Jun-11 | 21-Jun-11 | 28-Jun-11 | 29-Jun-11 | 15-Jul-11 |
